# Adjunctive Dexamethasone Affects the Expression of Genes Related to Inflammation, Neurogenesis and Apoptosis in Infant Rat Pneumococcal Meningitis

**DOI:** 10.1371/journal.pone.0017840

**Published:** 2011-03-11

**Authors:** Cornelia Blaser, Matthias Wittwer, Denis Grandgirard, Stephen L. Leib

**Affiliations:** 1 Institute for Infectious Diseases, University of Bern, Bern, Switzerland; 2 Spiez Laboratory, Spiez, Switzerland; University Freiburg, Germany

## Abstract

*Streptococcus pneumoniae* is the most common pathogen causing non-epidemic bacterial meningitis worldwide. The immune response and inflammatory processes contribute to the pathophysiology. Hence, the anti-inflammatory dexamethasone is advocated as adjuvant treatment although its clinical efficacy remains a question at issue. In experimental models of pneumococcal meningitis, dexamethasone increased neuronal damage in the dentate gyrus. Here, we investigated expressional changes in the hippocampus and cortex at 72 h after infection when dexamethasone was given to infant rats with pneumococcal meningitis. Nursing Wistar rats were intracisternally infected with *Streptococcus pneumoniae* to induce experimental meningitis or were sham-infected with pyrogen-free saline. Besides antibiotics, animals were either treated with dexamethasone or saline. Expressional changes were assessed by the use of GeneChip® Rat Exon 1.0 ST Arrays and quantitative real-time PCR. Protein levels of brain-derived neurotrophic factor, cytokines and chemokines were evaluated in immunoassays using Luminex xMAP® technology. In infected animals, 213 and 264 genes were significantly regulated by dexamethasone in the hippocampus and cortex respectively. Separately for the cortex and the hippocampus, Gene Ontology analysis identified clusters of biological processes which were assigned to the predefined categories “inflammation”, “growth”, “apoptosis” and others. Dexamethasone affected the expression of genes and protein levels of chemokines reflecting diminished activation of microglia. Dexamethasone-induced changes of genes related to apoptosis suggest the downregulation of the Akt-survival pathway and the induction of caspase-independent apoptosis. Signalling of pro-neurogenic pathways such as transforming growth factor pathway was reduced by dexamethasone resulting in a lack of pro-survival triggers. The anti-inflammatory properties of dexamethasone were observed on gene and protein level in experimental pneumococcal meningitis. Further dexamethasone-induced expressional changes reflect an increase of pro-apoptotic signals and a decrease of pro-neurogenic processes. The findings may help to identify potential mechanisms leading to apoptosis by dexamethasone in experimental pneumococcal meningitis.

## Introduction

Treatment of bacterial meningitis (BM) with sulfonamids was successfully introduced in the 1930's and the advent of third generation cephalosporins further reduced the mortality rates [1]. Since then, improvements in treatment success are scarce and the mortality rate of BM still reaches 34% and up to 50% of the survivors suffer from neurologic sequelae [2], [3]. Among the different pathogens causing community-acquired meningitis in industrialized countries, *Streptococcus pneumoniae* accounts for the majority of cases and shows the highest mortality rate [1], [4], [5], [6].

Studies investigating the pathophysiology of BM revealed that not only the pathogen itself exerts harmful effects but also the pronounced immune response of the host [4], [7]. Neurological complications such as increased intracranial pressure, cerebral ischemia, brain edema formation or hydrocephalus can lead to a fatal outcome [7]. Histopathological assessments in experimental models as well as autopsy cases showed three forms of injury in the central nervous system: apoptosis occurs in the hippocampal dentate gyrus, necrosis is found in the cerebral cortex, and loss of type 1 neurons in the spiral ganglion. These forms of neuronal damage cause neurological sequelae such as learning deficits, seizure disorders and hearing impairments respectively [8], [9], [10].

In order to reduce the inflammatory reaction, the glucocorticoid (GC) dexamethasone (dex) is advocated in patients with BM in addition to antibiotic treatment. Implementation of adjuvant therapy with dex (10 mg IV, given every 6 hours for 4 days started before or with the first dose of parenteral antibiotics) reduced mortality rate and the proportion of patients with unfavorable outcomes in the Netherlands [11]. Worldwide however, a meta-analysis including 2029 individual patient data concluded that the benefit of dex in BM remains unproven [12].

In experimental models both, detrimental and beneficial effects of adjunctive dex have been observed. Dex increased the number of apoptotic cells in the hippocampal dentate gyrus of infant rats with pneumococcal meningitis (PM) and of rabbits with *Escherichia coli* or PM [13], [14], [15] and led to decreased learning performance [13]. In different animals models however, Dex was shown to have otoprotective effects in experimental pneumococcal meningitis in gerbils [16], [17] or rabbits [18], and to improve neurobehavioral performance in adult rats with group B streptococcal meningitis [19].

A study assessing the transcriptome in experimental PM identified Gene Ontology (GO) terms related to “neuron generation” and “nervous tissue development” to be overrepresented when comparing the hippocampus of infected vs. sham-infected rats at one and three days after infection [20]. While these processes were mostly downregulated at day one, they were largely upregulated at day three. Thus, in this experimental disease model, the late acute phase until about three days after infection may offer a window of opportunity for therapeutic interventions to support neuronal regeneration. Besides its anti-inflammatory effects, dex is also reported to decrease neural proliferation and to act pro-apoptotic on neural precursors and immature neurons [21], [22], [23]. Therapies that act anti-proliferative, including dex, may impede regeneration by neurogenesis when administered at this disease stage [21], [24].

In the present study we investigated the effect of dex on the gene expression profile of the hippocampus and cortex in an infant rat model of PM 3 days after infection. The rationale for performing the analysis at the specific time point lies in the fact that in patients with meningitis, antibiotic therapy and adjuvant therapy with dex is initiated at this disease stage, and we therefore focused our investigation on the impact of dex on the early regenerative processes in the hippocampus. In experimental models of BM, gene expression analysis revealed regenerative processes to predominate over inflammatory processes in the hippocampus at this phase of disease [20]. Using the GO terms of biological processes we identified genes regulated by dex which are associated with the most important processes. These findings help to understand the impact of dex on BM.

## Methods

### Ethics Statement

All animal studies were approved by the Animal Care and Experimentation Committee of the Canton of Bern, Switzerland (Nr. 26/07), and followed the Swiss national guidelines for the performance of animal experiments.

### Animal model

A well established infant rat model of PM was used [13], [25], [26], [27]. Eleven days old nursing Wistar rats (n = 32) were infected intracisternally with 10.0 µl sterile saline containing 1.4×10^6^±5.5×10^5^ colony forming units (cfu)/ml of *Streptococcus pneumoniae* serotype 3 which has been isolated from a patient with invasive disease. Sham-infection for control animals (n = 24) was done with an equal volume of sterile saline 0.85%. Cerebrospinal fluid (CSF) was obtained by intracisternal puncture at 18 h after infection and 5 µl were cultured in serial dilutions on blood-agar plates to assess bacterial load [13], [26]. Clinical assessment was done by weighing the animals and applying a scoring system at predetermined time points (24 h, 48 h and 72 h after infection) [27]. Animals with a clinical score lower than 2 were euthanized for ethical reasons.

Antibiotic and adjuvant treatment of previous experimental PM studies in infant rats was adopted [13], [26]. Antibiotic therapy was started 18 h after infection in all animals by administration of ceftriaxone twice a day (100 mg/kg body weight intraperitoneally). Infected and control animals were then randomized to receive either saline (0.85% sterile saline sc, tid, n = 16 for infected and n = 12 for control animals) or an equal volume of saline containing dex (0.7 mg/kg body weight sc, tid, n = 16 for infected and n = 12 for control animals).

The animals were sacrificed 72 h after infection with an overdose of pentobarbital. Animals were perfused via the left cardiac ventricle using ice-cold phosphate-buffered saline (PBS). Then the brain was removed. Hippocampus and cortex were dissected from one hemisphere in ice-cold PBS and placed in tubes containing 500 µl and 1000 µl RNAstable reagent, respectively (kindly provided by Prof. Dr. phil. nat. Rolf Jaggi, Department of Clinical Research, University of Bern, Switzerland). The samples were kept at 4°C overnight and subsequently stored at −20°C until RNA isolation. The contralateral hemisphere was fixed and cryosections were prepared. The sections were stained with cresyl violet and evaluated for apoptosis in the dentate gyrus and ischemic tissue damage to the cerebral cortex as described elsewhere [13].

### Experimental setup

The experiments were designed to yield samples of the hippocampus and cortex from 4 experimental groups. Samples of the hippocampus derived from control animals treated with saline (HCCS), from control animals treated with dex (HCCD), from infected animals treated with saline (HCIS) and from infected animals treated with dex (HCID). The samples of the cortex derived from the equal experimental groups: from control animals treated with saline (CXCS), from control animals treated with dex (CXCD), from infected animals treated with saline (CXIS) and from infected animals treated with dex (CXID).

### RNA isolation

Total RNA of hippocampus and cortex was isolated using the EZ1 RNA Universal Tissue Kit and the EZ1 BioRobot (Qiagen, Hombrechtikon, Switzerland) according to the manufacturer's protocol. The tissue samples were placed in a tube containing 750 µl and 3.0 ml QIAzol Lysis reagent for the hippocampus and cortex, respectively. Homogenization was done for 30 s with a rotor-stator homogenizer (TissueRuptor, Qiagen). After five minutes of incubation at room temperature, 750 µl of the hippocampus and cortex homogenates were transferred to a new tube and 150 µl of chloroform were added. The samples were shaken vigorously and incubated at room temperature for two minutes. For optimal phase separation, the samples were centrifuged at 12′000× g for 15 minutes at 4°C. For the magnetic-particles based purification with the EZ1 RNA Universal Tissue Kit, 300 µl of the topmost aqueous phase was used as starting material. The DNase digestion step suggested in the protocol was included. The RNA was eluted in 50 µl elution buffer. Total RNA was stored at −80°C until use for microarray hybridisation and quantitative real-time PCR (qPCR).

In order to assess the quality of the isolated RNA, the Agilent Bioanalyzer 2100 was used with the RNA 6000 Nano Assay Kit. The manufacturer's protocol was followed. The implemented RNA integrity number (RIN) was used to estimate the quality of total RNA and to detect potential degradation. The concentration of total RNA was determined by measuring the absorbance at 260 nm with a spectrophotometer.

### RNA processing for hybridisation on GeneChip® Rat Exon 1.0 ST Microarrays

Total RNA was processed following the GeneChip® Whole Transcript Sense Target Labelling Assay Manual, Version 4 (Affymetrix, Santa Clara, CA, USA). RNA and DNA concentrations were determined with a spectrophotometer. The quality control of rRNA-reduced RNA and fragmented DNA was performed with the Agilent Bioanalyser 2100.

The starting amount was 1.5 µg total RNA. rRNA reduction was performed and the quality of the resulting RNA was assessed after column-based purification. Then, the RNA was used to synthesize double-stranded cDNA which was in vitro transcribed to antisense cRNA. After cleanup, 10 µg cRNA was used to generate sense cDNA. The cRNA was hydrolysed with RNaseH and the cDNA was purified using columns. Of each sample, 5.5 µg cDNA was fragmented and subsequently verified for its quality. Fragmented cDNA was labelled with biotin and added to the hybridisation cocktail at a concentration of 25 ng/µl. The hybridisation cocktail was then injected into GeneChip® Rat Exon 1.0 ST Arrays and the arrays were incubated at 45°C on a rotator in the hybridization oven 640 for 17 h at 60 rpm. The arrays were washed and stained on a Fluidics Station 450 according to the Fluidics Procedure FS450_0001. The Arrays were processed with the GeneChip® Scanner 3000 7G. DAT image and CEL intensity files of the microarrays were generated using the Affymetrix GeneChip® Command Console (version 0.0.0.676, Affymetrix).

### Datamining of microarray data

All data is MIAME compliant and has been deposited in the ArrayExpress database of the European Bioinformatics Institute (http://www.ebi.ac.uk/arrayexpress, accession number E-MEXP-2953).

Data analysis was performed using the oneChannel Graphical User Interface (version 1.12.1, www.bioinformatica.unito.it/oneChannelGUI) [28] which is a package available in Bioconductor open source software for bioinformatics (www.bioconductor.org) [29] and implemented in the R Project for Statistical Computing (version 2.10.0, www.r-project.org) [30]. Quality control of the CEL files was done using Expression Console software (version 1.1, Affymetrix). All CEL files were deemed suitable for analysis and they were uploaded to oneChannelGUI using Affymetrix Power Tools (Version 1.12.0, www.affymetrix.com/partners_programs/programs/developer/tools/powertools.affx). The model-based Robust Multi-array Average (RMA) algorithm was used to generate the probe set summary based on the full annotation on gene- and exon-level [31].A filtering step was introduced and only genes that are expressed above a defined intensity threshold of 132.5 on 10% of the arrays were retained, i.e. at least on 3 of 28 arrays. The limma algorithm was used to compute a linear model fit [32]. For significance testing, the different groups within one tissue were compared one by one, e.g. HCID vs. HCIS or CXCD vs. CXCS. The Benjamini and Hochberg procedure was used to correct for multiple testing [33]. Genes with a false discovery rate less than 0.05 were considered as differentially expressed. Only genes with a unique Entrez Gene identifier (ID) were used for further analysis.

#### Correspondence Analysis (COA)

As previously described, a COA was done to reduce the data to variables which can be displayed in a three-dimensional graphic [20]. The analysis was based on log_2_-intensities.

#### GO analysis of biological processes

The functional annotation clustering tool of the Database for Annotation, Visualization, and Integrated Discovery (DAVID, david.abcc.ncifcrf.gov) was used to search for clusters of biological processes of the GO database within two sets of differentially expressed genes, namely HCID vs. HCIS and CXID vs. CXIS (http://david.abcc.ncifcrf.gov) [34], [35]. The list of all genes expressed above the intensity threshold with an ID was used as background. The default annotation category for biological processes of GO (GOTERM_BP_FAT) and the medium classification stringency settings were selected. GO clusters with an enrichment score greater than 1.3 were considered significant [35], [36]. These clusters were grouped to predefined generic categories which were “inflammation”, “growth”, “apoptosis”, “immune system development”, “signalling”, “hypoxia”. Multiple GO clusters were not assigned to one of these categories due to lack of coherence.

### RNA processing for qPCR using TaqMan Low Density Arrays

Reverse transcription was performed with 1.5 ng total RNA using the High-Capacity cDNA Reverse Transcription Kit including the RNase inhibitor (Applied Biosystems, Foster City, CA). The manufacturer's protocol was followed. An additional RT-negative reaction without adding the transcriptase was run for each sample using one third of RNA input and volumes. The reaction volume was 20 µl and the standard cycling protocol was used.

RT-positive and -negative cDNA (75 ng) was controlled for successful reverse transcription and the absence of genomic DNA with TaqMan Gene Expression Assays (Applied Biosystems). The three genes *glyceraldehyde-3-phosphate dehydrogenase* (GAPDH), *transmembrane protein* (Tmem) 111 and *vesicle docking protein USO1 homolog* (USO1) were used in PCR. The manufacturer's protocol was followed. TaqMan Gene Expression Master Mix was used and the standard cycling protocol was run.

The expression of 48 selected genes was assessed in qPCR using TaqMan Low Density Arrays (Applied Biosystems). Pre-designed TaqMan Gene Expression Assays were chosen to generate a customized array. The manufacturer's protocol was followed and the arrays were run on a 7900HT Real-Time PCR System operated by the Sequence Detection Systems software (Version 2.3, Applied Biosystems). The input was 200 ng of cDNA for each sample. The TaqMan Universal PCR Master Mix was used and the standard cycling protocol was followed. Four arrays were run and the data were combined using the RQ Manager software (Version 1.2, Applied Biosystems). Raw C_q_ values were exported for further analysis.

### Data analysis of qPCR

The qPCR results were analysed using the ΔΔC_q_ method implemented in the online tool RT^2^ Profiler PCR Array Data Analysis (http://www.sabiosciences.com/pcr/arrayanalysis.php; SABiosciences, Frederick, MD, USA). No adjustment for primer efficiency was done because all pre-designed TaqMan Gene Expression Assays have been thoroughly tested by Applied Biosystems and are declared to have equivalent amplification efficiencies close to hundred percent [37]. The cut-off C_q_ was 35 cycles and to normalize gene expression, the geometric mean of the reference genes was used. GAPDH as a widely used reference gene was adopted and supplemented by USO1, Tmem111 and ribosomal protein L24 (Rpl24). The supplemental reference genes were selected based on the data of a previous microarray study in infant rats suffering from PM [20], [38]. As suggested in previous work, genes that showed a coefficient of variance of the raw C_q_ values greater than 4% were excluded from further evaluation [39].

### Assessments on the protein level

#### Protein isolation

In order to obtain tissue samples for protein extraction, the same procedures as for RNA extraction were followed on the experimental model of meningitis. Additionally, CSF was sampled by intracisternal puncture at 26 h and 72 h after infection. After the dissection of hippocampus and cortex, the tissue sample was weighed and supplemented with seven times as much PBS (1∶7 m∶v) containing 0.1% Triton-X 100, Complete Protease Inhibitor Cocktail Tablets ® and phosphatase inhibitors, i.e. 1× Phosphatase Inhibitor Cocktail 1 ® (both from Roche Diagnotics AG, Rotkreuz, Switzerland), 1 mM sodium orthovanadate, 1 mM sodium fluoride, 20 mM sodium pyrophosphate and 2 mM beta-glycerophosphate. The tissue was homogenized and the suspension incubated on ice for 20 min. After centrifugation at 4°C and 16′000× g for 5 min, the supernatant was collected and stored at −80°C until further processing.

#### Measurement of protein concentration

The protein concentration was measured with the bicinchoninic acid (BCA) protein assay (Thermo Fisher Scientific Inc., Rockford, IL, USA). Bovine serum albumin in concentrations between 0 and 2000 µg/ml was used as standard curve. The hippocampus and cortex samples were diluted 1∶50 and 1∶100 respectively with PBS containing 0.1% Triton-X 100. Then, 25 µl sample or standard were added with 200 µl working reagent in duplicates to a microplate. Incubation was done at 37°C for 30 min. The colorimetric measurement at 550 nm was done with a microplate reader at room temperature. Protein concentrations were calculated with the implemented “Protein Quantification with BCA” protocol in SoftMax Pro software (Version 5.3, Molecular Devices).

#### Western blotting

Proteins were separated with a 10% sodium dodecylsulfate polyacrylamide gel electrophoresis. Sample, 4x sample buffer containing 5% beta-mercaptoethanol and water were mixed to a final protein concentration of 50 µg and a volume of 20 µl. Denaturing was done at 95°C for 5 min and was followed by electrophoresis at 100 V for 1 h and 50 min. The separated proteins were transferred to a Polyvinylidene fluoride membrane (0.45 µm) in a tank transfer system with transfer buffer (25 mM Tris(hydroxymethyl)-aminomethan (Tris), 192 mM glycine, 20% methanol and 0.05% SDS) at 90 V for 2 h. The membrane was washed with Tris-buffered saline containing 0.05% Tween 20 (TBST) and blocked for 1 h with 5% milk in TBST. Incubation with the primary antibody against protein *tyrosine phosphatase, non-receptor type 6* (PTPN6) (1∶500 in TBST; 68 kDa; 3759S, Cell Signaling Technology, Danvers, MA, USA) was done at 4°C overnight. The membrane was washed and incubated for 1 h at room temperature with the secondary antibody coupled to peroxidase (1∶20′000 in TBST; A1949, Sigma-Aldrich). Chemiluminescent detection was performed for 5 min with luminal as horseradish peroxidase substrate. Protein signals were detected by exposure of the membrane to medical X-ray films and developed with an image processor. The membrane was stripped at 37°C for 20 min. A wash step followed and the reprobing procedure started at the blocking step. The primary antibody against beta-actin (1∶10′000 in TBST; 42 kDa; A5316, Sigma-Aldrich) and its corresponding secondary antibody (1∶20′000 in TBST; A9309, Sigma-Aldrich) were used.

#### Luminex® xMAP® technology

MILLIPLEX MAP kits (Millipore Corporation) were used for microsphere-based multiplex immunoassays to measure the concentration of different proteins [25]. Brain-derived neurotrophic factor (BDNF) was measured in homogenates of the hippocampus and cortex using a single-plex Rat Pituitary Kit. The following cytokines and chemokines were measured in CSF at 26 h and 72 h after infection, and tissue homogenates using a ten-plex Rat Cytokine Kit: Interleukin 1 beta (IL-1b), IL-2, IL-6, IL-10, chemokine C-C motif ligand 2 (CCL2), CCL3, CCL5, chemokine C-X-C motif ligand 10 (CXCL10) interferon gamma (IFN-g) and tumor necrosis factor alpha (TNF-a). The cytokines/chemokines assessed were selected for analysis based on results from previous studies in the experimental model [25], [40]. The manufacturer's protocol of each kit was followed. PBS containing 0.1% Triton-X 100 and 5 mg/ml bovine serum albumin was used as matrix. CSF samples were prepared in a fivefold dilution and the tissue homogenates were standardized to the smallest protein concentration detected in the homogenates. At least 100 beads were counted per analyte. Analysis of raw data was done with the BIO-PLEX manager software (Version 4.1, BioRad Laboratories, Hercules, CA, USA). Statistical analyses were done with the GraphPad Prism software (Version 5.01, GraphPad Software, La Jolla, CA). The non-parametric Kruskal-Wallis test was used to compare all groups within the time point or tissue. When this test was significant, infected and dex-treated animals were compared to infected and saline-treated animals using the Mann Whitney test.

## Results

### Animal model

The CSF samples of the infected animals showed positive cultures for *Streptococcus pneumoniae* at 18 h after infection documenting successful infection (1.2×10^6^ cfu/ml–1.3×10^8^ cfu/ml). No difference between ID vs. IS was observed. The infected animals showed clinical signs of disease reflected by weight loss (24 h, 48 h and 72 h after infection) and reduced clinical scores (24 h and 72 h after infection) (Table 1). Five infected animals of both treatment groups were euthanized because they showed a clinical score lower than 2. Animals with PM showed a significantly reduced weight in comparison to sham-infected animals (p<0.001 using the unpaired t test for all time points). Dex significantly decreased the weight of control animals at all time points and in infected animals only at 72 h after infection (p<0.01 using the unpaired t test). In order to induce a uniform disease with low mortality, a moderate disease severity was induced which did not result in ischemic tissue damage to the cerebral cortex at 72 h after infection. The number of apoptotic cells was not significantly different when comparing ID vs. IS (p = ns using the Mann Whitney U test).

**Table 1:** Overview of the clinical parameters of all experimental groups.

Animal status	Weight difference at 24 h pi [g]	Weight difference at 48 h pi [g]	Weight difference at 72 h pi [g]	Clinical score at 24 h pi	Clinical score at 72 h pi	Number of apoptotic cells
Control + saline (n = 7)	3.4±0.5	5.1±1.2	6.4±2.5	5.0±0.0	5.0±0.0	0.1 [0.0–0.5]
Control + dexamethasone (n = 7)	2.3±0.5	2.1±0.4	2.6±1.3	5.0±0.0	5.0±0.0	2.2 [0.2–3.9]
Infected + saline (n = 8)	−0.5±1.1	−2.1±1.7	−0.4±2.0	4.0±0.8	3.9±0.4	0.1 [0.0–0.2]
Infected + dexamethasone (n = 8)	−1.3±0.9	−3.6±1.0	−3.1±1.5	3.9±0.4	3.9±0.4	0.0 [0.0–0.4]

The number of animals include all animals used for the microarray experiment only or used for the microarray experiment and the qPCR analysis. Mean ± standard deviation are given for weight difference and clinical score, median and [range] are given for the number of apoptotic cells. pi = post infection.

### RNA isolation

All RNA samples fulfilled the minimal quality requirements of a RIN>7.0 [41], [42], [43]. Based on the high quality of the isolated RNA we excluded samples with a RIN<8.0. The RIN of the samples used for microarray hybridization was 8.7±0.3 (mean ± SD) and it was 8.8±0.2 for the samples used in qPCR analysis.

### RNA processing for hybridisation on GeneChip® Rat Exon 1.0 ST Microarrays

The RIN and the weight difference of the animals at 48 h after infection were used as parameters to reduce variability from the starting material. Outliers regarding the weight showed weight gain in infected animals or no weight gain in control animals. Based on this criterion, one animal per group was excluded from further selection. Six samples (2× CXIS; 1× CXID; 2× CXCS and 1× HCCS) showed a RIN lower than 8 and were therefore excluded. From the remaining samples, 4 hippocampal and cortical samples of ID and IS animals were randomly chosen while 3 samples were randomly chosen of ID and IS animals.

In order to increase array detection sensitivity and specificity, rRNA reduction was performed. Evaluation of the Bioanalyzer 2100 plots showed appropriate quality for all samples at both in-process checkpoints during RNA preparation, i.e. after rRNA reduction and fragmentation of DNA.

### Datamining of microarray data

#### Overview

**Figure 1:**
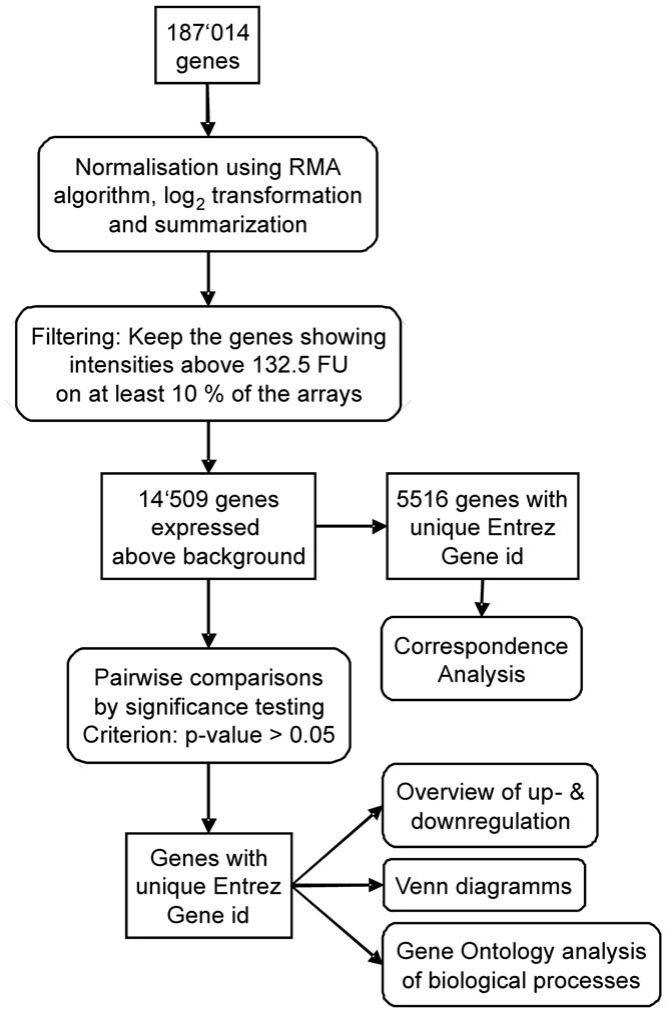
Workflow of the gene expression analysis of the microarray data. Square textboxes represent the gene lists at the different steps of the workflow and round-edged textboxes represent the different steps performed for data analysis. RMA = Robust Multi-array Average, FU = fluorescence units, id = identifier.

**Figure 2:**
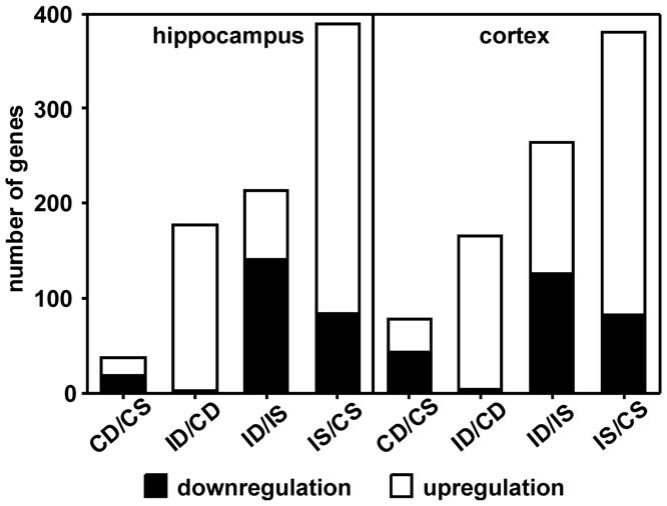
The numbers of significantly regulated genes by comparing the different treatment groups. C = control animals, D = dexamethasone treatment, S = saline treatment, I = infected animals.

Raw and normalized data are available in the ArrayExpress database of the European Bioinformatics Institute (http://www.ebi.ac.uk/arrayexpress, accession number E-MEXP-2953). 187′014 probesets were available for analysis when the full annotation option was selected for upload of the data to oneChannelGUI (Figure 1). The number of probesets was reduced to 14′509 by removing probesets expressed below the defined background threshold. After applying the linear model fit, paired comparisons of experimental groups resulted in the following numbers of significantly up- and downregulated genes with an identifier (Figure 2). The highest number of genes was regulated when comparing IS vs. CS, i.e. 390 genes in the hippocampus and 380 genes in the cortex. Of these genes, 78% were upregulated in both tissues. Approximately half as many genes changed their expression when comparing ID vs. CD. In this comparison, 98% of 177 genes were upregulated in the hippocampus and 97% of 166 genes were upregulated in the cortex. The comparison of ID vs. IS revealed expressional changes in 213 genes in the hippocampus and 264 genes in the cortex showing 34% and 52% upregulation respectively. The least genes were regulated when comparing CD vs. CS.

#### Venn diagrams

**Figure 3:**
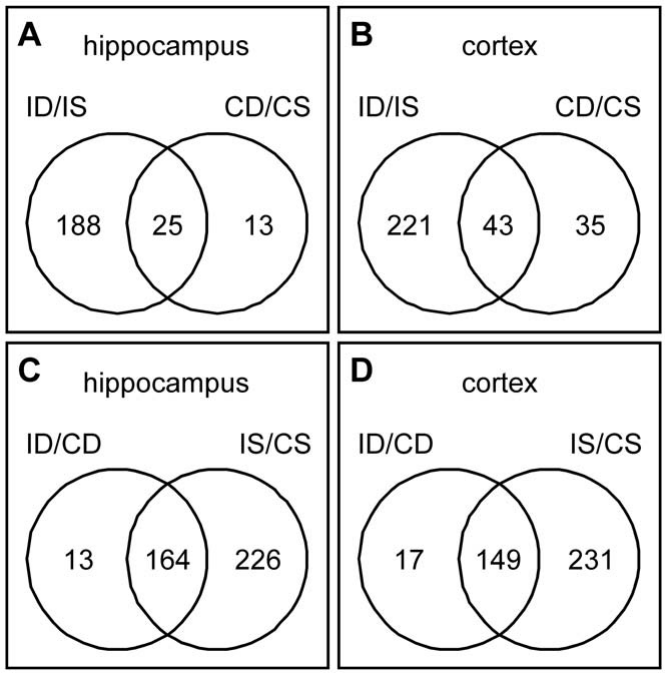
Venn diagrams showing the relation of the number of genes of defined comparisons. The numbers in the left or right part of the circles represent the number of regulated genes in the given comparison. The number in the intersection of both circles represents the number of genes regulated in both comparisons. The influence of the treatment in infected vs. control animals within the hippocampus (A) and cortex (B) is shown. The intersection shows the number of genes which are regulated by the treatment irrespective of the infection. The influence of the infection in dexamethasone vs. saline treated animals within the hippocampus (C) and cortex (D) is shown. The intersection shows the number of genes regulated by the infection irrespective of the treatment. I = infected animals, D = dexamethasone treatment, S = saline treatment, C = control animals.

Venn diagrams help to understand the relation of regulated genes between different given comparisons. They allow identifying the number of genes regulated by multiple comparisons. When looking for the effect of the treatment, 25 genes in the hippocampus and 43 genes in the cortex were regulated by dex regardless of the infection status (Figure 3). 188 genes in the hippocampus and 221 genes in the cortex changed their expression only when infected animals were treated with dex. It was also shown that 164 genes in the hippocampus and 149 genes in the cortex were regulated by the infection irrespective of the treatment.

#### COA

**Figure 4:**
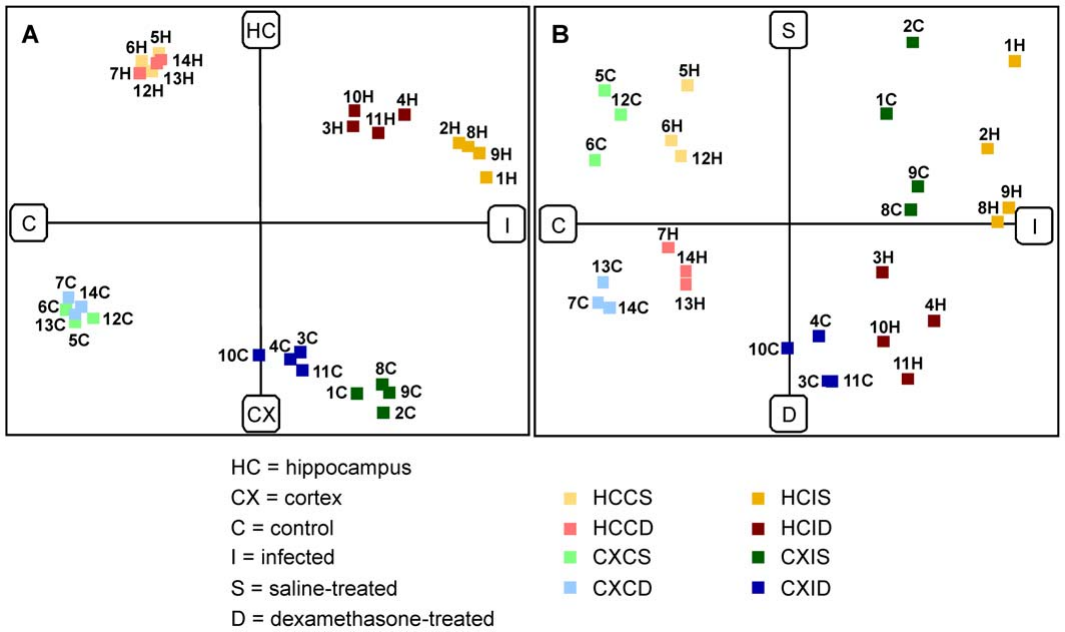
The correspondence analysis (COA) of the microarray dataset. The COA separated in component one (x axis in A and B) the control from the infected animals, in component two (y axis in A) the hippocampus from the cortex samples and in component three (y axis in B) the dexamethasone treated from the saline treated animals. Each square represents an array. H = hippocampus, C = cortex. Equal numbers in the hippocampus and cortex group indicates that these samples derived from the same animal.

The COA reduced the multidimensional microarray data to the three most informative components describing the dataset. Samples that are close to one another on the plot show similar expression profiles. All experimental groups were separated from each other and the respective samples clustered together (Figure 4). The first component that explains most of the variance in the data set separated infected animals and control animals. The second component discriminated between hippocampus and cortex samples. The third component separated dex-treated and saline-treated animals. These three components found in unsupervised dimension reduction reflect the experimental setup and validate the dataset.

#### GO analysis

In the hippocampus, the functional annotation clustering tool of DAVID generated 68 GO clusters of biological processes out of the differentially regulated genes when comparing ID vs. IS. Twenty-five of these GO clusters showed an enrichment score greater than 1.3. In the cortex, 81 GO clusters were formed but only 15 of them showed an enrichment score greater than 1.3.

**Figure 5:**
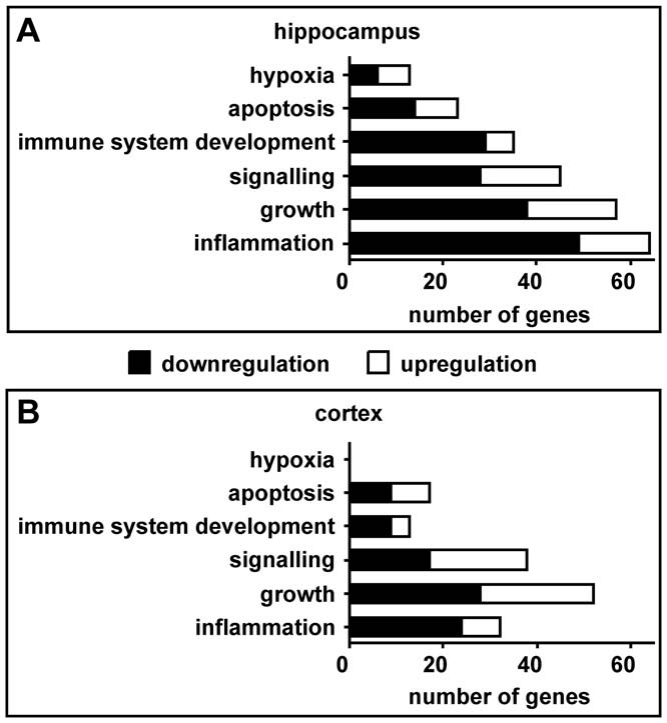
Gene Ontology (GO) analysis of biological processes. Infected and dexamethasone-treated animals were compared with infected and saline-treated animals (ID vs. IS) in the hippocampus (A) and the cortex (B). GO clusters of biological processes were generated by the functional annotation clustering tool of the Database for Annotation, Visualization, and Integrated Discovery (DAVID). These clusters were then assigned to six defined categories.

The genes associated to specific GO clusters were then summarized in six defined categories (Tables S1 and Table S2). The three categories with the highest numbers of regulated genes were “inflammation”, “growth” and “signalling” in both tissues (Figure 5). “Inflammation” included the highest number of genes in the hippocampus while it included less genes than “growth” and “signalling” in the cortex. More regulated genes were found in “growth” than in “signalling” in both tissues. With the exception of the category “signalling” in the cortex, all these categories included more down- than upregulated genes. Likewise, more down- than upregulation was observed in the categories “immune system development” and “apoptosis”. While “immune system development” included more regulated genes than “apoptosis” in the hippocampus, the latter one included more genes than “immune system development” in the cortex. The category “hypoxia” included the least regulated genes in the hippocampus and was not represented in the cortex.

### RNA processing for qPCR using TaqMan Low Density Arrays

The 14 samples from the hippocampus used for microarray hybridisation were also analyzed by qPCR analysis. Per group, four additional hippocampal samples from littermates were run in qPCR (n = 8 for infected animals, n = 7 for sham-infected animals).

Successful reverse transcription was confirmed by the quality check of the cDNA using the assays for the reference genes GAPDH, USO1 and Tmem111. All reverse transcribed samples were detected by all three PCR reactions. Of the three reference genes, solely GAPDH is able to detect genomic DNA since its primers are positioned within a single exon. Genomic DNA was detected in multiple RT-negative samples, but the C_q_ value was greater than 35 which was defined as sensitivity threshold for qPCR evaluation. Furthermore, the C_q_ value of the RT-negative sample was more than 15 times greater than the one in the RT-positive sample indicating a difference in DNA amount of a factor of 30′000. Hence, the amount of genomic DNA was considered negligible.

### Data analysis of qPCR

One sample of the infected and dex-treated animals was excluded from the analysis based on irregular qPCR plots. GAPDH was initially used as one of four reference genes. However, its use as reference gene was questioned in the literature lately [38], [39]. Likewise we observed fluctuations of GAPDH expression within our experimental setup. Therefore we excluded it from the list of reference genes. Two genes, namely CXCL10 (ID 245920) and *POU class 5 homeobox 1* (POU5f1, ID 294562) were not analysed due to a coefficient of variance greater than 4%.

**Figure 6:**
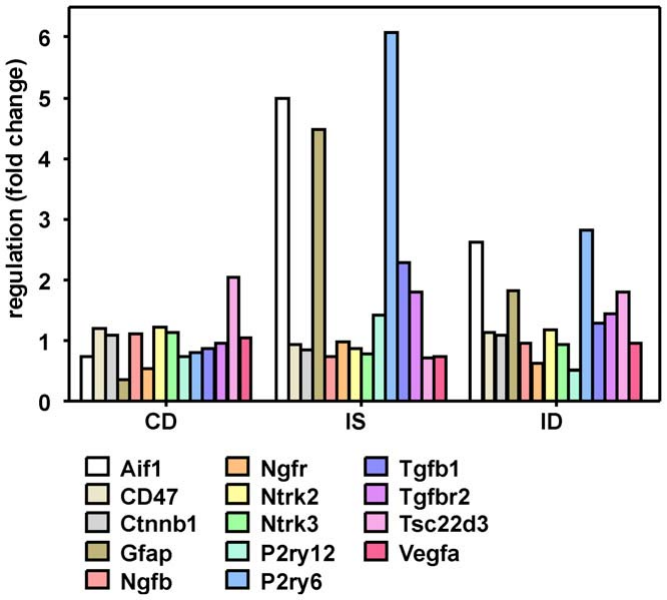
Gene expression pattern of the quantitative real-time PCR analysis in the hippocampus. The three experimental groups were compared to control and saline-treated animals. These 14 genes were significantly regulated when comparing infected and dexamethasone-treated animals with infected and saline-treated animals (ID vs. IS). C = control animals, D = dexamethasone treatment, I = infected animals, S = saline treatment.

In a first step, HCID vs. HCIS were compared (Table S3). The fold changes of qPCR and microarray analysis showed a highly significant Spearman correlation of r = 0.804 (p<0.0001). Fourteen genes were found to be significantly regulated in qPCR (Table 2). In order to assess the expressional pattern of these genes, the three experimental groups HCCD, HCIS and HCID were compared to the control group HCCS (Figure 6). Two expressional patterns separated these genes. Genes downregulated by the infection and upregulated by dex were CD47 (ID 29364), *Catenin (cadherin associated protein) beta 1* (Ctnnb1, ID 84353), *nerve growth factor* (NGF, ID 310738), *neurotrophic tyrosine kinase receptor type 2* (NTRK2, ID 25054) and NTRK3 (ID 29613), *TSC 22 domain family member 3* (TSC22d3, ID 83514) and *vascular endothelial growth factor* (VEGFA, ID 83785). The inversed pattern was observed for the *allograft inflammatory factor 1* (AIF-1, ID 29427), the *glial fibrillary acidic protein* (GFAP, ID 24387), the NGF receptor (NGFR, ID 24596), the *purinergic G-protein coupled receptor P2Y 12* (P2Y12, ID 64803), the *pyrimidinergic G-protein coupled receptor P2Y 6* (P2Y6, ID 117264), the *transforming growth factor beta 1* (TGF-b1, ID 59086) and the *TGF-b receptor 2* (Tgfbr2, ID 81810).

**Table 2:** Results of the significantly regulated genes on TaqMan Low Density Arrays.

Gene description (Gene symbol)	TaqMan Assay id	Reference Sequence	Fold change qPCR ID/IS	CI (95%) qPCR ID/IS	Fold change MA ID/IS
Allograft inflammatory factor 1 (AIF-1)	Rn00567906_g1	NM_017196.2	0.526	0.347–0.705	0.648
Glial fibrillary acidic protein (GFAP)	Rn00566603_m1	NM_017009.2	0.406	0.269–0.544	0.466
Nerve growth factor, beta (NGF)	Rn01533872_m1	XM_227525.3	1.310	1.125–1.495	1.088
Neurotrophic tyrosine kinase, receptor, type 2 (NTRK2)	Rn01441749_m1	NM_012731.1	1.380	1.243–1.517	1.171
Neurotrophic tyrosine kinase, receptor, type 3 (NTRK3)	Rn00570389_m1	NM_019248.1	1.191	1.057–1.326	1.042
Nerve growth factor receptor (NGFR)	Rn00561634_m1	NM_012610.1	0.628	0.447–0.810	1.032
Catenin (cadherin associated protein), beta 1 (Ctnnb1)	Rn00584431_g1	NM_053357.2	1.296	1.121–1.471	1.046
Transforming growth factor, beta 1 (TGF-b1)	Rn99999016_m1	NM_021578.2	0.565	0.445–0.686	0.635
Transforming growth factor, beta receptor II (Tgfbr2)	Rn00579682_m1	NM_031132.3	0.800	0.698–0.902	0.752
CD47 antigen (CD47)	Rn00569914_m1	NM_019195.2	1.223	1.115–1.332	1.039
Purinergic receptor P2Y, G-protein coupled 12 (P2Y12)	Rn02133262_s1	NM_022800.1	0.351	0.250–0.452	0.497
Pyrimidinergic receptor P2Y, G-protein coupled, 6 (P2Y6)	Rn02134326_s1	NM_057124.2	0.464	0.319–0.610	0.544
TSC22 domain family 3 (TSC22d3)	Rn00580222_m1	NM_031345.1	2.512	2.095–2.929	1.790
Vascular endothelial growth factor A (VEGFA)	Rn01511602_m1	NM_031836.2	1.305	1.050–1.559	1.370

id = identifier, qPCR = quantitative real-time PCR, ID = infected and dexamethasone-treated animals, IS = infected and saline-treated animals, CI = confidence interval, MA = microarray. A fold change below one shows downregulation, a fold change above one shows upregulation.

### Assessments on the protein level

#### Western blotting

**Figure 7:**
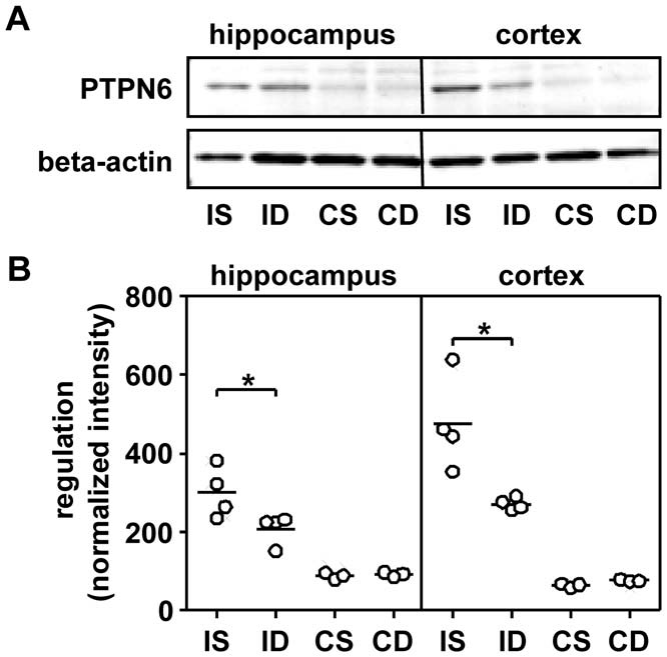
Western blot and microarray assessment of tyrosine phosphatase, non-receptor type 6 (PTPN6). The western blot analysis showed increased protein levels in hippocampus and cortex homogenates of infected animals compared to control samples (A). This finding confirms the results of the microarray experiment (B). I = infected animals, S = saline treatment, D = dexamethasone treatment, C = control animals.

Analysis of PTPN6 (ID 116689) expression on the mRNA level showed an increase by the infection and a reduction by dex in infected animals. These findings were confirmed on the protein level by western blot analysis (Figure 7).

#### Luminex® xMAP® Technology

The protein measurements in hippocampus and cortex homogenates showed significant decreases of CCL2, CCL3 and CCL5 when comparing ID vs. IS (Table 3). No changes by dex were observed in the levels of the other chemokines and cytokines assessed. BDNF was shown to be constantly higher expressed in the hippocampus than in the cortex. Within the same tissue, no differences in BDNF protein levels were observed.

**Table 3:** Results of the multiplex immunoassays in tissue homogenates (A) and/or cerebrospinal fluid (B).

A)	Hippocampus [pg/mg total protein] (72 h pi)					Cortex [pg/mg total protein] (72 h pi)				
Protein	CS (n = 4)	CD (n = 4)	IS (n = 5)	ID (n = 7)	Ratio (ID/IS)	CS (n = 4)	CD (n = 4)	IS (n = 5)	ID (n = 7)	Ratio (ID/IS)
MIP-1a/CCL3	1.70	0.48	6.69	1.76	0.26 *	0.27	0.70	3.78	0.91	0.24 *
MCP-1/CCL2	5.43	8.84	8.84	5.01	0.57 *	3.27	4.77	7.44	3.51	0.47 *
RANTES/CCL5	7.40	8.05	39.59	15.63	0.39 *	6.13	7.10	25.32	10.88	0.43 *
IP-10/CXCL10	-	-	-	-	-	-	-	-	-	-
TNF-a	5.91	7.12	5.93	4.49	0.76	3.38	3.97	2.63	2.24	0.85
IFN-g	-	-	3.57	5.03	1.41	-	-	1.84	2.28	1.24
IL-1b	16.13	13.60	19.28	12.92	0.65	6.92	6.94	17.37	8.59	0.49
IL-2	37.51	39.56	16.80	14.77	0.88	17.57	22.03	15.15	8.69	0.57
IL-6	24.98	18.72	16.35	16.97	1.04	14.77	12.22	8.22	10.54	1.28
IL-10	7.16	6.91	4.60	3.26	0.71	4.18	3.82	2.31	2.52	1.09
BDNF (n = 6 for “C”)	52.89	45.53	27.05	59.26	2.19	5.60	4.52	4.88	9.12	1.87

B)	26 h pi in CSF [pg/ml]					72 h pi in CSF [pg/ml]				
Protein	CS (n = 5)	CD (n = 5)	IS (n = 5)	ID (n = 3)	Ratio (ID/IS)	CS (n = 5)	CD (n = 5)	IS (n = 4)	ID (n = 3)	Ratio (ID/IS)
MIP-1alpha/CCL3	-	-	466.27	536.12	1.15	-	-	-	-	-
MCP-1/CCL2	-	-	15656.19	22391.21	1.43	-	-	654.70	214.34	0.33
RANTES/CCL5	188.74	292.38	1559.93	2601.10	1.67	251.52	314.88	790.91	434.15	0.55
IP-10/CXCL10	121.77	113.08	113.08	183.67	1.62	152.03	137.64	43.97	121.77	2.77 *
TNF-a	-	-	134.49	231.65	1.72	-	-	6.18	14.33	2.32
IFN-g	-	-	40396.90	36139.64	0.89	-	-	186.50	50.83	0.27 *
IL-1b	11.60	11.60	230.18	439.00	1.91	11.60	22.48	11.60	11.60	1.00
IL-2	18.35	18.35	73.78	121.40	1.65	29.83	18.35	37.96	18.36	0.48
IL-6	-	-	48328.63	28767.47	0.60	-	-	-	-	-
IL-10	-	-	2577.83	3846.96	1.49	-	-	-	-	-

Results are given as median. “-” indicates values that were below detection limit. * p<0.05 was considered as significantly different. pi = post infection, C = control animals, S = saline treatment, D = dexamethasone treatment, I = infected animals, CSF = cerebrospinal fluid.

Protein levels in the CSF showed a significant increase in CXCL10 expression when comparing ID vs. IS at 72 h after infection (Table 3). In contrast, a decrease in IFN-g levels by dex was seen in CSF at 72 h after infection.

## Discussion

Conducting a microarray experiment yields an enormous amount of raw data. Appropriate statistical methods are required to evaluate such data sets and the use of biological databases helps to circumvent interpretational pitfalls. Due to the limited number of samples, i.e. four and three samples for infected and sham-infected animals respectively, the control of the sample variability is essential. Differences can be introduced by the starting material and the technical processing.

In the experimental model of meningitis, clinical parameters such as clinical score and weight difference were assessed at defined time points. In order to decrease variability within the starting samples, the weight difference at 48 h after infection was used. Animals with an outlier value of weight difference were excluded, e.g. weight gain in infected animals or weight loss in control animals. In order to process only RNA showing high quality, samples with an RIN lower than 8.0 were removed. Three control animals and 4 infected animals per treatment were chosen from the remaining samples for the microarray experiment. Four additional hippocampal samples of littermates per group were used for qPCR analysis in order to increase the number of samples.

The technical processing was monitored by in-process quality controls of the samples during the synthesis of targets for microarray hybridization. In order to control the hybridization step, the raw data were evaluated for their accuracy based on intensity and expression box plots, MA plots and spike-in controls. All samples passed the different quality check points and were used for data mining. A COA was performed after the probe summarization in order to visualize the structure of the expressional data (Figure 4). The samples were correctly separated by the three parameters infection, brain region and treatment. First, this result shows that fundamental differences evoked by these three parameters do exist and second, that the variability between the different groups is sufficiently low to allow the separation of the groups.

Another issue to be addressed when doing a whole genome expression analysis of brain tissue is the complex functional and anatomical structure of the brain [20], [44]. Different cell types (e.g. neurons, glial cells and subtypes within) form various brain areas or are grouped within the same area into structurally distinct subregions. Moreover, two challenges arise in the evaluation of expression profiling studies by the heterogeneous nature of brain tissue. Biologically important differences are often due to small changes in gene expression [44]. Therefore, the typically used cut-off level, i.e. greater than a two-fold change, is not appropriate for expression profiling in brain tissue which poses the discrimination of real differences from experimental noise as a significant issue. The other challenge lies within the relative amount of different cell types [44]. A significant fold change of one gene can be diluted when occurring in a cell type representing only a fraction of the overall cell population being studied. Furthermore, downregulation of a gene in one cell population and upregulation of this gene in a neighbouring cell population results in an underestimation of the real change.

Microglial cells are the brain resident macrophages of the central nervous system (CNS). When activated, they play an important role in both the innate immunity and the pathogenesis of CNS infections such as BM [45]. Inflammatory mediators and nitric oxide released by microglial cells can contribute to neuronal damage. Besides this direct induction of apoptotic death of neurons, microglial cells indirectly cause neuronal damage by attracting monocytes and neutrophils. The detrimental effects manifest on neural stem and progenitor cells, the precursors of neurons localized in the subgranular zone of the hippocampal dentate gyrus [46], [47]. It is this brain region that shows apoptotic cell death in experimental BM. In the infant rat model of PM the extent of apoptosis is further enhanced by dex, an observation that underlies the present study [13]. Dex as a GC exerts anti-inflammatory effects and inhibits the expression of pro-inflammatory molecules. Additionally, dex has been shown to suppress proliferation of neuronal cells [21], [22], [23].

The impact of dex on inflammation, damage and regeneration in experimental PM is discussed below with the emphasis on the hippocampus, a brain structure where these processes overlap. Therefore, only significant expressional changes when comparing HCID vs. HCIS will be discussed based on genes assigned to the categories “inflammation”, “apoptosis” and “growth” found in GO cluster analysis of biological processes (Table 4). A significant difference of gene expression when comparing CXID vs. CXIS is mentioned when observed.

**Table 4:** Fold changes of significantly different genes on microarrays which are mentioned in the discussion.

Entrez Gene id	Gene description (symbol)	Fold change ID/IS hippocampus	Fold change ID/IS cortex
117029	chemokine (C-C motif) receptor 5 (CCR5)	0.329	0.332
171056	chemokine (C-X3-C) receptor 1 (CX3CR1)	0.383	0.414
24387	glial fibrillary acidic protein (GFAP)	0.466	0.451
287526	serine (or cysteine) peptidase inhibitor, clade F, member 1 (Serpinf1)	0.488	-
64803	purinergic receptor P2Y, G-protein coupled 12 (P2Y12)	0.497	-
25124	signal transducer and activator of transcription 1 (STAT1)	0.517	0.553
287435	Cd68 molecule (CD68)	0.532	-
29591	transforming growth factor, beta receptor 1 (Tgfbr1)	0.533	0.556
58962	prostaglandin D2 synthase 2, hematopoietic (HPGDS)	0.572	-
116689	protein tyrosine phosphatase, non-receptor type 6 (PTPN6)	0.585	-
54259	inositol polyphosphate-5-phosphatase D (INPP5D)	0.639	0.694
24404	glutathione peroxidase 1 (Gpx1)	0.667	0.694
308444	Axl receptor tyrosine kinase (Axl)	0.678	0.746
57027	a disintegrin and metallopeptidase domain 17 (ADAM17)	0.700	-
81810	transforming growth factor, beta receptor II (Tgfbr2)	0.752	-
303786	apoptosis-inducing factor, mitochondrion-associated 3 (Aifm3)	2.341	2.205
24484	insulin-like growth factor binding protein 3 (IGFBP-3)	1.932	2.222
83514	TSC22 domain family 3 (TSC22d3)	1.790	2.069
81809	transforming growth factor, beta 2 (TGF-b2)	1.711	1.420
29610	transforming growth factor, beta receptor III (Tgfbr3)	1.525	1.578
81811	thrombopoietin (THPO)	1.336	-
25054	neurotrophic tyrosine kinase, receptor, type 2 (NTRK2)	-	1.254

id = identifier, ID = infected and dexamethasone treated animals, IS = infected and saline treated animals. A fold change below one shows downregulation, a fold change above one shows upregulation.

### Inflammation

GCs are the most commonly used anti-inflammatory and immunosuppressive drugs in a variety of inflammatory and immune diseases. The anti-inflammatory effect is based on the activation of anti-inflammatory gene expression and the repression of pro-inflammatory gene expression [48]. Dex is a synthetic GC which shows a 20 to 30 times higher potency to evoke anti-inflammatory effects relative to the endogenously produced cortisol. Dex was reported to inhibit the activation of microglia which was confirmed in the qPCR results showing a significant downregulation of AIF-1, a marker of activated microglia [45], [49]. Its expression was found to be increased already in the early acute phase of experimental BM and peaked at 3 days after infection [2], [20]. Immunohistochemical detection confirmed the expressional data and showed the morphological change of microglia from the resting and ramified shape to the active and amoeboid form [20], [45]. In a rat model of traumatic brain injury, dex was able to decrease the accumulation of AIF-1-positive cells [49]. CD68 antigen (CD68, ID 287435) is a marker for microglial cells that have differentiated into a macrophage-like phenotype [20], [45]. The downregulation of CD68 by dex in PM confirms the inhibition of microglial activation. GFAP, which is another glial marker, assigns the activation of astrocytes and was downregulated by dex. GFAP expression was upregulated in the cortex and hippocampus of animals with PM [2], [20]. Astrocytes were shown to rescue neurons from microglial glutamate-induced death by the uptake of exogenous glutamate via excitatory amino acid transporters [50]. The excitatory amino acid glutamate was found to be elevated in the CSF of patients with BM [51]. In addition, glutamate influx into the peri- and endolymph was suggested to be a possible link between blood-labyrinth barrier disruption and hearing loss in BM [52].

Inhibition of microglia activation prevents the production of chemokines and cytokines [45]. In hippocampus and cortex homogenates of infected animals we detected significant decreases by dex on the protein level of *chemokine C-C motif ligand 2* (CCL2, ID 24770), CCL3 (ID 25542) and CCL5 (ID 81780). These proteins were reported to be released by microglia when stimulated with pneumococcal cell wall [53]. CCL2 is a monocyte chemoattractant protein and recruits macrophages and microglia to inflammatory sites [54]. CCL2 was observed to be induced in the acute phase of PM [55]. Dex was shown to inhibit the production of CCL2 mRNA and protein by activated microglia resulting in the inhibition of microglial migration [56]. CCL3 was associated to neutrophil recruitment while CCL5 plays a role in recruiting leukocytes to inflammatory sites [54]. In the CSF of infected animals, CXCL10 was upregulated on the protein level by dex at 72 h after infection. The protein was found to be induced at 24 h after infection with *Streptococcus pneumoniae* but was no longer detected later on [55]. CXCL10 is also known as the IFN-g-inducible protein. However, we found decreased IFN-g (ID 25712) protein levels in CSF by dex. Results of the analysis on the level of gene expression differed from those on the protein level, indicating that cytokine/chemokines are modulated at both levels similar to what has been observed in other experimental paradigms [57].

*Chemokine C-X3-C motif receptor 1* (CX3CR1, ID 171056) was downregulated by dex in the cortex and the hippocampus. It is a G-protein-coupled receptor localized on microglial and immune cells. Its ligand fractalkine is neuronally expressed and may be involved in the activation and chemoattraction of microglia into injured tissue following ischemia [58]. CX3CR1 was shown to be downregulated in the early acute phase (22 h after infection) and upregulated in the late acute phase (44 h after infection) of BM [2].

P2Y12 was shown to be downregulated by dex in the hippocampus by microarray and qPCR assessment. The latter one additionally detected a downregulation of P2Y6 by dex. P2Y12 is a receptor at which nucleotides, mainly ATP, induce microglial chemotaxis towards sites of neuronal damage at early stages of local CNS injury [59], [60], [61]. The receptor shows robust expression in resting microglia which is reduced after microglial activation. Lack of P2Y12 in knockout mice revealed a significant delay but no complete abolishment of microglial chemotaxis [60]. When hippocampal neurons were damaged in vivo and in vitro, an increase in P2Y6 mRNA co-localizing with activated microglia was observed [62]. The receptor is activated by endogenous UDP that was leaked by injured neurons triggering microglial phagocytosis [59], [61], [62].

*Signal transducer and activator of transcription 1* (STAT1, ID 25124) was downregulated by dex in the hippocampus. It was observed to be continuously upregulated in the acute phase of BM [2]. STAT1 is directly activated by reactive oxygen species within ischemic neurons and regulates the cellular response to IFN-g [63]. It was shown that dex inhibits STAT1 expression when activated by IFN-g in peripheral blood mononuclear cells [64]. The observed reduction of IFN-g protein level by dex in experimental PM may add to the decrease of STAT1 expression.

*ADAM metallopeptidase domain 17* (ADAM17, ID 57027) was downregulated by dex in the present study. It proteolytically releases the pro-inflammatory TNF-a that triggers the secretion of matrix metalloproteinases (MMPs) which in turn are involved in blood brain barrier disruption [27]. Combined inhibition of ADAM17 and MMPs in experimental models of BM resulted in the attenuation of brain damage, preservation of learning performance and reduced frequency of seizures [27], [65].

Expressional data show a reduction of *haematopoietic prostaglandin D synthase* (HPGDS, ID 58962) by dex. HPGDS is a key enzyme in the production of prostaglandin D [66]. It is markedly induced after focal cerebral ischemia followed by reperfusion and is mainly produced by microglia. HPGDS protects against the detrimental effects of cerebral infarction.

We observed a downregulation of *serine or cysteine peptidase inhibitor clade F member 1* (Serpinf1, ID 287526) by dex. The protein belongs to the family of serine protease inhibitors. It exerts neurotrophic effects on retinal and hippocampal cell neurons and acts as a pro-inflammatory agent [67]. Serpinf1 changes microglial morphology to a more deactivated state and induces production of CCL3 and its receptors *chemokine C-C motif receptor 3* (CCR3, ID: 117027) and CCR5 (ID 117029), the latter one being downregulated by dex on gene level. On protein level, we showed a decrease of two ligands of CCR5 in tissue homogenates, namely CCL3 and CCL5. *In vivo* it was shown that Serpinf1 is effective in protecting CNS neurons from ischemic insult [68].

TSC22d3 was upregulated by dex in the hippocampus of infected animals. The protein was shown to be expressed in different cell types including neurons of the hippocampus [69], [70], [71]. The expression was shown to be induced by GCs and the protein mediates their anti-inflammatory effects [69], [70]. For instance, in bone marrow mesenchymal stem cells it was shown that TSC22d3 inhibits inflammatory-induced COX-2 expression by blocking NF-kappaB nucluear translocation [70].

### Growth - Neurogenesis

The cellular and functional impairment of the hippocampus following BM and its modulation by dex does not exclusively result from regulation of genes associated with the category “apoptosis”. Genes belonging to “growth” may likewise determine the fate of neuronal cells and decide on survival, neurogenesis or death.

We observed an upregulation of *thrombopoietin* (THPO, ID 81811) by dex in the hippocampus. In rat hippocampal neurons, THPO induced cell death and the induction of pro-apoptotic proteins most likely contributed to this effect [72]. Studies in PC12 cells indicated that THPO decreases neuronal survival by suppressing NGF-induced ERK activity [73]. When primary hippocampal neuronal cultures were challenged with hypoxia, THPO decreased on mRNA and protein level [74]. Furthermore, *Axl receptor tyrosine kinase* (Axl, ID 308444) was downregulated by dex in the hippocampus. Axl was shown to be upregulated by NGF in PC12 cells and in association with the NTRK1 it supports neuronal differentiation and survival [75].

The neurotrophin NGF and the NTRK2 (ID 25054) and NTRK3 (ID 29613) were found to be upregulated by dex in qPCR assessment while NGFR was downregulated. The differences were not detected in the microarray data which might be due to the limited number of samples used and the lower sensitivity of microarrays. Neurotrophin signalling through NTRK receptors can regulate cell survival, proliferation, the fate of neural precursors, axon and dendrite growth [76]. NGF determines neuronal survival by suppressing NGFR-induced apoptotic signalling and by initiating survival through the activation of NTRK1 [77]. As will be described in more detail in the apoptosis section, PTPN6 can dephosphorylate NTRK1 thus abolishing the pro-survival signal of increased NGF expression. BDNF (ID 24225) and *neurotrophin 5* (NTF5, ID 25730) are specific to NTRK2 while NT3 mainly activates NTRK3 [78]. Each neurotrophin also binds to NGFR which activates either synergistic or antagonistic processes to those activated by NTRK binding [76], [78]. In the present study, no significant differences of neurotrophin mRNA or BDNF protein level at three days after infection were detected when comparing ID vs. IS. At five days after infection however, BDNF mRNA and protein levels were increased by dex treatment [79]. The observed upregulation of BDNF mRNA and protein already at four days after infection suggests that dex is able to delay the increase of BDNF [80]. Thus the previously observed beneficial effects of BDNF on neuronal damage in BM may be delayed if not even abolished by dex treatment [81].

We found expressional regulation of genes by dex in the hippocampus which are involved in the pro-neurogenic TGF-b signalling pathway. In the microarray data, an upregulation of Tgfbr3 (ID 29610) and of TGF-b2 (ID 81809) by dex was observed, while the Tgfbr1 (ID 29591) and Tgfbr2 (ID 81810) were downregulated. qPCR analysis confirmed the downregulation of Tgfbr2 by dex and showed furthermore a decrease of TGF-b1. In adrenalectomized rats, as a correlate for GC depletion, TGF-b1 expression was increased in the hippocampus and correlated with the significant proliferation of newborn neurons [82]. In addition, TGF-b1 exerts neuroprotection by the induction of the transcriptional activity of NF-kappaB which in turn promotes anti-apoptotic effects in cultured hippocampal neurons [83]. Thus, we suggest that the downregulation of TGF-b1 and the receptors 1 and 2 by dex results in a lack of pro-neurogenic signalling via TGF-b pathway and a reduced induction of anti-apoptotic gene transcription both resulting in increased apoptosis.

Ctnnb1 was observed to be upregulated in the hippocampus by dex. The protein is involved in the canonical branch of the pro-neurogenic Wnt signalling pathway [84], [85]. When Ctnnb1 translocates to the nucleus, a complex is formed with transcription factors of the T-cell factor/lymphoid enhancer factor family to activate target genes [85]. In rat mammary epithelial tumor cells, dex was able to upregulate Ctnnb1 transcript and protein expression and caused the protein to localize predominantly at the cell membrane rather than the nucleus [86]. Likewise, dex was shown to decrease the cytosolic amount of Ctnnb1 and to inhibit the nuclear translocation of Ctnnb1 both resulting in a suppression of the Wnt signal in cultured human osteoblasts [85]. Studies in adult dentate gyrus derived neural precursors showed opposite regulation of proliferation by dex and lithium through contrariwise intranuclear translocation of Ctnnb1 [21]. The mood stabilizer lithium was shown to induce adult hippocampal progenitors to become neurons dependent on Wnt pathway components such as an elevation of Ctnnb1 [84], [85].

### Apoptosis

During BM, apoptosis is observed in the hippocampal dentate gyrus. The occurrence of cells showing characteristics of apoptosis peaks at 36 h after infection and declines to control levels until 72 h after infection [87]. In the present study which focuses on regenerative processes subsequent to apoptosis, the animals were sacrificed at 72 h after infection. Histopathological evaluation showed the highest number of apoptotic cells in sham infected control animals treated with dex. No significant difference was observed when comparing ID vs. IS. We hypothesize, that cells vulnerable to the pro-apoptotic effects of dex undergo apoptosis and subsequent phagocytosis and clearing before 72 h as a result of the infection while the apoptotic cascade induced by dex treatment starts later in sham-infected control animals to the result that apoptotic cells are still visible at 72 h. This view is supported by the finding of upregulation of genes that were associated to the category “apoptosis” when comparing HCID vs. HCIS suggesting that dex prolongs processes of apoptosis in infected animals.

*Glutathione peroxidase 1* (Gpx1, ID 24404) was downregulated by dex in the cortex and hippocampus and it was observed to be upregulated during the acute phase of BM [2]. In Gpx1 knockout mice, increased neuronal cell death was demonstrated following cerebral ischemia-reperfusion injury [88]. Studies in primary neuronal cells lacking Gpx1 also showed increased levels of cell death following the addition of exogenous hydrogen peroxide. This correlated with a downregulation of the activation of the phospho-inositide 3-kinase (PI3K) – Akt survival pathway [88]. In contrast, *inositol polyphosphate 5′ phosphatase* (INPP5D, ID 54259), which was downregulated by dex in experimental PM, can negatively regulate the PI3K signalling by hydrolization of the second messenger PI-3,4,5-trisphosphate [89].

An upregulation of *insulin-like growth factor binding protein 3* (IGFBP-3, ID 24484) was observed in the hippocampus by dex. Studies on the protein level showed an induction in brain tissue throughout early and late acute phase of PM in mice [55]. The protein was shown to elicit anti-proliferative and pro-apoptotic effects in an array of different cell systems [90].

Dex treatment increased *mitochondrial apoptosis-inducing factor 3* (Aifm3, ID 303786) mRNA levels in the hippocampus and cortex. Earlier studies revealed that the increase of apoptosis during PM is not exclusively due to the activation of caspases. The bacteria itself were able to damage mitochondria in microglia and neurons which was followed by the release of Aifm3 [91]. Increased expression by dex may reflect enhanced apoptosis.

PTPN6 was upregulated by the infection and downregulated by dex in the hippocampus of infected animals. Western blot analysis confirmed the results found on the microarrays. PTPN6 was found to be expressed in astrocytes, microglia and neurons of the hippocampus [77], [92]. Cortical lesions led to an upregulation of PTPN6 in activated microglia and astrocytes [92]. In PC12 cells and neurons, PTPN6 dephosphorylated NTRK1 (ID 59109) resulting in apoptosis [77].

### Conclusion

By gene expression profiling, we documented the downregulation of inflammatory processes by adjunctive dex treatment in experimental PM. We detected diminished expression of genes involved in the activation and migration of microglial cells resulting in the observed reduction of chemokines on protein level. Microglia are involved in the initial immune response against PM by recognizing bacterial components via the toll-like receptors 2 and 4 subsequently releasing inflammatory mediators [45], [93]. Furthermore, we observed a downregulation of ADAM17 which is involved in TNF-a processing and blood brain barrier disruption occurring in BM.

Dex treatment affected the expression of genes and pathways regulating “apoptosis” or “growth” in experimental PM. The expressional changes by dex lead to an enhancement of pro-apoptotic processes or to a lack of pro-survival signals. These findings may help to identify the mechanisms underlying the observed increased apoptosis and learning difficulties by adjuvant dex in experimental PM.

## Supporting Information

Table S1 **Microarray results of significantly different genes when comparing samples of the hippocampus of infected and dexamethasone-treated animalswith infected and saline-treated animals (ID vs. IS).**
(DOC)

Table S2 **Microarray results of significantly different genes when comparing samples of the cortex of infected and dexamethasone-treated animalswith infected and saline-treated animals (ID vs. IS).**
(DOC)

Table S3 **Results of all genes used on TaqMan Low Density Arrays.**
(DOC)
