# Supplementary material for: Adjunctive Dexamethasone Affects the Expression of Genes Related to Inflammation, Neurogenesis and Apoptosis in Infant Rat Pneumococcal Meningitis
Source: PLoS One. 2011 Mar 11;6(3):e17840. doi: 10.1371/journal.pone.0017840 (PMC3055894; doi:10.1371/journal.pone.0017840)
Supplement: Table S1 — Microarray results of significantly different genes when comparing samples of the hippocampus of infected and dexamethasone-treated animalswith infected and saline-treated animals (ID vs. IS). (DOC) [file pone.0017840.s001.doc]

**Supporting table S1. Microarray results of significantly different genes when comparing samples of the hippocampus.**

| **Entrez Gene id** | **Gene description** | **Fold change ID/IS** | **Categories of GO clusters** |
| --- | --- | --- | --- |
| 498335 | chemokine (C-X-C motif) ligand 13 | 0.054 | IS dev |
| 171164 | guanylate nucleotide binding protein 2 | 0.258 |  |
| 316137 | EGF-like module containing, mucin-like, hormone receptor-like sequence 1 | 0.275 |  |
| 246208 | interferon gamma inducible protein 47 | 0.284 |  |
| 24575 | myxovirus (influenza virus) resistance 1 | 0.307 |  |
| 117029 | chemokine (C-C motif) receptor 5 | 0.329 | inf |
| 24932 | CD4 antigen | 0.369 | inf, IS dev, sig |
| 25291 | annexin A3 | 0.376 |  |
| 171056 | chemokine (C-X3-C) receptor 1 | 0.383 | apo, inf, IS dev |
| 308995 | integrin alpha L | 0.386 | growth, inf, IS dev, sig |
| 292594 | leukocyte immunoglobulin-like receptor, subfamily B, member 4 | 0.388 |  |
| 304966 | Fc fragment of IgG, low affinity IIIa, receptor | 0.395 | IS dev |
| 155918 | lymphocyte cytosolic protein 2 | 0.396 | inf, IS dev |
| 24699 | protein tyrosine phosphatase, receptor type, C | 0.396 | apo, growth, inf, IS dev, sig |
| 303090 | immunity-related GTPase family, M | 0.403 |  |
| 315348 | NCK associated protein 1 like | 0.408 |  |
| 497942 | chemokine (C-X-C motif) ligand 16 | 0.419 | growth, inf |
| 24615 | S100 calcium-binding protein A4 | 0.419 |  |
| 497009 | N-acylsphingosine amidohydrolase (acid ceramidase)-like (predicted) | 0.420 |  |
| 366988 | bridging integrator 2 | 0.422 |  |
| 362792 | phospholipase D family, member 4 | 0.427 |  |
| 25423 | cathepsin C | 0.430 |  |
| 366957 | RAS-related C3 botulinum substrate 2 | 0.432 | growth, inf |
| 25383 | apolipoprotein B editing complex 1 | 0.433 | inf |
| 114553 | neutrophil cytosolic factor 1 | 0.439 | inf |
| 171452 | RAB3A interacting protein (rabin3)-like 1 | 0.451 |  |
| 24387 | glial fibrillary acidic protein | 0.466 |  |
| 24968 | proteasome (prosome, macropain) subunit, beta type 8 (large multifunctional peptidase 7) | 0.477 | sig |
| 292060 | interferon regulatory factor 8 | 0.479 | inf, IS dev, sig |
| 287526 | serine (or cysteine) peptidase inhibitor, clade F, member 1 | 0.488 | growth, inf |
| 303735 | ring finger protein 213 | 0.488 |  |
| 304549 | 2'-5' oligoadenylate synthetase-like 2 | 0.496 |  |
| 364674 | TRAF-interacting protein with forkhead-associated domain, family member B | 0.496 |  |
| 64803 | purinergic receptor P2Y, G-protein coupled 12 | 0.497 | growth, IS dev |
| 362456 | Rho, GDP dissociation inhibitor (GDI) beta | 0.499 |  |
| 24811 | transporter 1, ATP-binding cassette, sub-family B (MDR/TAP) | 0.501 | inf, IS dev |
| 64552 | macrophage expressed gene 1 | 0.501 |  |
| 298199 | adipose differentiation related protein | 0.507 |  |
| 171411 | transmembrane protein 176B | 0.508 |  |
| 25124 | signal transducer and activator of transcription 1 | 0.517 | apo, growth, inf, sig |
| 297077 | transmembrane protein 176A | 0.522 |  |
| 288077 | hematopoietic cell specific Lyn substrate 1 | 0.525 | growth, IS dev, sig |
| 29185 | CD37 antigen | 0.530 | inf, IS dev |
| 287435 | Cd68 molecule | 0.532 |  |
| 29591 | transforming growth factor, beta receptor 1 | 0.533 | apo, growth, inf, hypoxia, IS dev, sig |
| 89783 | lysosomal-associated protein transmembrane 5 | 0.533 |  |
| 25059 | hexokinase 2 | 0.540 |  |
| 113955 | glycoprotein (transmembrane) nmb | 0.546 | growth, inf |
| 155183 | src family associated phosphoprotein 2 | 0.547 | IS dev |
| 81515 | Yamaguchi sarcoma viral (v-yes-1) oncogene homolog | 0.564 | growth, inf, IS dev, sig |
| 25181 | biglycan | 0.570 | growth |
| 58919 | cyclin D1 | 0.572 | growth, sig |
| 58962 | prostaglandin D2 synthase 2, hematopoietic | 0.572 | inf |
| 338477 | src-like adaptor | 0.575 |  |
| 140860 | solute carrier organic anion transporter family, member 2b1 | 0.579 |  |
| 310392 | solute carrier family 7 (cationic amino acid transporter, y+ system), member 11 | 0.579 |  |
| 293650 | T-cell, immune regulator 1, ATPase, H+ transporting, lysosomal V0 protein A3 | 0.584 |  |
| 307212 | carnosine dipeptidase 1 (metallopeptidase M20 family) | 0.584 |  |
| 116689 | protein tyrosine phosphatase, non-receptor type 6 | 0.585 | apo, inf, IS dev, sig |
| 406864 | chloride intracellular channel 1 | 0.585 |  |
| 192262 | complement component 1, s subcomponent | 0.586 | inf, IS dev |
| 54320 | podoplanin | 0.591 | growth, inf, hypoxia, IS dev |
| 25441 | Fc fragment of IgE, high affinity I, receptor for; gamma polypeptide | 0.597 | apo, growth, inf, IS dev |
| 302248 | nidogen 2 | 0.603 | inf |
| 25668 | CD38 antigen | 0.607 | growth, inf, hypoxia, IS dev |
| 81521 | moesin | 0.610 | inf |
| 63865 | legumain | 0.611 | growth |
| 24251 | CD53 antigen | 0.613 |  |
| 29143 | granulin | 0.614 | growth |
| 289668 | leucine aminopeptidase 3 | 0.615 |  |
| 301097 | loss of heterozygosity, 11, chromosomal region 2, gene A homolog (human) | 0.617 |  |
| 81756 | RAB13, member RAS oncogene family | 0.621 |  |
| 308906 | similar to 9230105E10Rik protein | 0.622 |  |
| 25021 | integrin alpha M | 0.627 | inf, IS dev |
| 306071 | lymphocyte cytosolic protein 1 | 0.630 |  |
| 59073 | latexin | 0.631 |  |
| 54259 | inositol polyphosphate-5-phosphatase D | 0.639 | apo, inf, IS dev |
| 25663 | interleukin 1 receptor, type I | 0.645 |  |
| 25737 | proliferating cell nuclear antigen | 0.645 |  |
| 25406 | CD44 antigen | 0.648 | apo,growth, inf |
| 304023 | ST3 beta-galactoside alpha-2,3-sialyltransferase 6 | 0.648 |  |
| 300870 | family with sequence similarity 46, member A | 0.648 |  |
| 85424 | E74-like factor 1 | 0.648 | inf, sig |
| 292808 | peptidase D | 0.653 |  |
| 293118 | prolylcarboxypeptidase (angiotensinase C) | 0.654 |  |
| 498333 | BMP-2 inducible kinase | 0.656 | sig |
| 288774 | signal transducer and activator of transcription 2 | 0.659 |  |
| 84398 | complement component 1, q subcomponent, receptor 1 | 0.661 | inf |
| 305604 | epidermal growth factor-containing fibulin-like extracellular matrix protein 1 | 0.664 |  |
| 24404 | glutathione peroxidase 1 | 0.667 | apo, growth, inf, IS dev, sig |
| 64519 | ectonucleoside triphosphate diphosphohydrolase 1 | 0.667 | IS dev |
| 252832 | zinc finger CCCH type, antiviral 1 | 0.669 | inf |
| 360903 | fibroblast growth factor receptor-like 1 | 0.671 |  |
| 295629 | CD302 antigen | 0.676 |  |
| 308444 | Axl receptor tyrosine kinase | 0.678 | growth, inf, sig |
| 116685 | lamin B1 | 0.679 |  |
| 114861 | serine carboxypeptidase 1 | 0.681 |  |
| 313210 | ATP-binding cassette, sub-family A (ABC1), member 1 | 0.686 | inf |
| 310378 | nicotinamide nucleotide transhydrogenase | 0.692 |  |
| 54227 | actin related protein 2/3 complex, subunit 1B | 0.696 | growth |
| 25484 | myosin IE | 0.700 | growth, IS dev, sig |
| 57027 | a disintegrin and metallopeptidase domain 17 | 0.700 | apo, growth, inf, hypoxia, IS dev, sig |
| 361383 | CD97 antigen | 0.701 |  |
| 83515 | ROD1 regulator of differentiation 1 (S. pombe) | 0.702 |  |
| 81513 | ligase I, DNA, ATP-dependent | 0.702 |  |
| 304860 | N-acetylneuraminate pyruvate lyase | 0.704 |  |
| 282838 | GM2 ganglioside activator protein | 0.712 | inf, sig |
| 289014 | MAP kinase-activated protein kinase 2 | 0.715 | sig |
| 317218 | integral membrane protein 2A | 0.718 |  |
| 24924 | pleiotrophin | 0.718 | growth, inf |
| 58982 | neurocan | 0.719 | inf |
| 25745 | myosin, heavy polypeptide 9, non-muscle | 0.721 | growth, inf, IS dev |
| 25625 | tumor necrosis factor receptor superfamily, member 1a | 0.726 | inf, sig |
| 363269 | nuclear antigen Sp100 | 0.728 | growth, sig |
| 246249 | Rho GTPase activating protein 4 | 0.733 | growth |
| 25359 | thymopoietin | 0.734 |  |
| 353307 | solute carrier family 29 (nucleoside transporters), member 3 | 0.740 |  |
| 362519 | structural maintenance of chromosomes 2 | 0.741 |  |
| 54319 | villin 2 | 0.741 | inf |
| 679870 | calpain, small subunit 2 | 0.742 |  |
| 312652 | plexin D1 | 0.744 | growth |
| 65168 | secretory carrier membrane protein 2 | 0.746 |  |
| 311171 | leucine rich repeat containing 55 | 0.748 |  |
| 81810 | transforming growth factor, beta receptor II | 0.752 | growth, inf, hypoxia, IS dev, sig |
| 29474 | coronin, actin-binding protein, 1B | 0.758 |  |
| 25293 | aquaporin 4 | 0.760 |  |
| 306817 | DEK oncogene (DNA binding) | 0.761 |  |
| 83718 | chloride intracellular channel 4 (mitochondrial) | 0.763 | growth |
| 24842 | transformation related protein 53 | 0.764 | apo, growth, inf, hypoxia, IS dev, sig |
| 24356 | v-ets erythroblastosis virus E26 oncogene homolog 1 (avian) | 0.765 | apo, growth, inf, sig |
| 266813 | paired related homeobox 1 | 0.767 | growth, sig |
| 114122 | versican | 0.768 | growth, inf |
| 313130 | peptidase M20 domain containing 2 | 0.770 |  |
| 307505 | catenin (cadherin associated protein), alpha 1 | 0.771 | apo, growth, inf, sig |
| 494338 | tripartite motif-containing 25 | 0.781 |  |
| 312705 | complement component 1, r subcomponent | 0.782 |  |
| 64529 | cathepsin B | 0.793 |  |
| 29528 | vesicle-associated membrane protein 3 | 0.795 | inf |
| 306825 | myosin regulatory light chain interacting protein | 0.803 | inf |
| 81664 | guanine nucleotide binding protein, alpha inhibiting 2 | 0.803 | growth, sig |
| 29492 | notch gene homolog 2 (Drosophila) | 0.815 | apo, growth, sig |
| 361734 | membrane-spanning 4-domains, subfamily A, member 4A | 7.120 |  |
| 83783 | sulfotransferase family 1A, phenol-preferring, member 1 | 6.278 |  |
| 64345 | hypoxia inducible factor 3, alpha subunit | 3.636 | hypoxia, sig |
| 60666 | glycerol-3-phosphate dehydrogenase 1 (soluble) | 2.913 |  |
| 84403 | gap junction membrane channel protein beta 6 | 2.595 | growth |
| 65164 | HtrA serine peptidase 1 | 2.584 |  |
| 295228 | G patch domain containing 4 | 2.561 |  |
| 117240 | hephaestin | 2.487 | IS dev |
| 24450 | 3-hydroxy-3-methylglutaryl-Coenzyme A synthase 2 | 2.378 |  |
| 303786 | apoptosis-inducing factor, mitochondrion-associated 3 | 2.342 | apo, sig |
| 408223 | ubiquitin specific peptidase 54 | 2.025 |  |
| 361810 | FK506 binding protein 5 | 2.001 |  |
| 29624 | arachidonate 5-lipoxygenase activating protein | 1.958 | inf |
| 312382 | ATP-binding cassette, sub-family G (WHITE), member 2 | 1.944 |  |
| 24701 | phosphorylase, glycogen, muscle | 1.937 | hypoxia |
| 24484 | insulin-like growth factor binding protein 3 | 1.933 | apo, growth, sig |
| 24716 | ret proto-oncogene | 1.917 | growth, inf, sig |
| 497811 | xanthine dehydrogenase | 1.889 |  |
| 140942 | DNA-damage-inducible transcript 4 | 1.883 | hypoxia |
| 25116 | hydroxysteroid 11-beta dehydrogenase 1 | 1.860 | growth |
| 94174 | tubulointerstitial nephritis antigen-like 1 | 1.826 |  |
| 83514 | TSC22 domain family 3 | 1.790 | apo, inf |
| 25037 | myelin-associated oligodendrocytic basic protein | 1.778 |  |
| 25420 | crystallin, alpha B | 1.745 | apo, growth, sig |
| 81809 | transforming growth factor, beta 2 | 1.711 | apo, growth, inf, hypoxia, IS dev, sig |
| 24191 | aldolase C | 1.674 | growth, hypoxia |
| 316275 | progestin and adipoQ receptor family member VIII | 1.670 |  |
| 290655 | cytokine receptor-like factor 1 | 1.658 |  |
| 246246 | patatin-like phospholipase domain containing 7 | 1.644 |  |
| 60423 | solute carrier family 28 (sodium-coupled nucleoside transporter), member 2 | 1.611 |  |
| 24250 | cystathionine beta synthase | 1.604 | growth, sig |
| 315189 | proline rich 5 (renal) | 1.580 |  |
| 298504 | major facilitator superfamily domain containing 2 | 1.575 |  |
| 54250 | fibroblast growth factor 2 | 1.555 | apo, growth, inf, sig |
| 64032 | connective tissue growth factor | 1.531 | growth, inf, sig |
| 29610 | transforming growth factor, beta receptor III | 1.525 | growth, hypoxia, IS dev, sig |
| 361749 | interleukin 33 | 1.485 |  |
| 353227 | zinc finger and BTB domain containing 16 | 1.476 | apo, growth, IS dev, sig |
| 60356 | cysteine sulfinic acid decarboxylase | 1.461 |  |
| 25566 | tropomodulin 1 | 1.460 |  |
| 300517 | similar to hypothetical protein FLJ25530 | 1.439 |  |
| 171522 | cytochrome P450, family 2, subfamily d, polypeptide 4 | 1.436 | inf |
| 307759 | glutamic pyruvate transaminase (alanine aminotransferase) 2 (predicted) | 1.436 |  |
| 59326 | Rap guanine nucleotide exchange factor (GEF) 3 | 1.419 | sig |
| 363085 | carbonic anyhydrase 12 | 1.413 |  |
| 94172 | solute carrier family 27 (fatty acid transporter), member 1 | 1.389 | inf |
| 64511 | farnesyltransferase, CAAX box, beta | 1.383 | growth, sig |
| 299923 | N-myc downstream regulated gene 1 | 1.381 | IS dev |
| 114024 | acyl-CoA synthetase long-chain family member 3 | 1.379 | inf |
| 81632 | 4-aminobutyrate aminotransferase | 1.363 | inf, hypoxia |
| 498728 | similar to Elongation of very long chain fatty acids protein 2 | 1.343 |  |
| 171114 | N-myc downstream regulated gene 2 | 1.342 |  |
| 116486 | SEC14-like 2 (S. cerevisiae) | 1.340 |  |
| 81811 | thrombopoietin | 1.336 | growth, inf, IS dev |
| 498545 | TSC22 domain family, member 1 | 1.334 |  |
| 287422 | period homolog 1 (Drosophila) | 1.311 |  |
| 56817 | Kv channel-interacting protein 2 | 1.305 |  |
| 290566 | oxoglutarate dehydrogenase-like | 1.304 |  |
| 171410 | acyl-CoA synthetase bubblegum family member 1 | 1.303 | inf |
| 81651 | chondroitin sulfate proteoglycan 4 | 1.301 | growth, inf, sig |
| 24791 | secreted acidic cysteine rich glycoprotein | 1.301 | growth, inf |
| 361129 | hyaluronan and proteoglycan link protein 4 | 1.300 | inf |
| 117560 | Kruppel-like factor 9 | 1.284 | growth |
| 25435 | eukaryotic elongation factor-2 kinase | 1.283 | sig |
| 83537 | sphingomyelin phosphodiesterase 2, neutral | 1.281 | apo |
| 294303 | tetraspan transmembrane protein, hair cell stereocilia | 1.267 | growth |
| 362518 | similar to RIKEN cDNA 2810432L12 | 1.259 |  |
| 81530 | pyruvate dehydrogenase kinase, isoenzyme 2 | 1.241 | sig |
| 293489 | GIY-YIG domain containing 2 | 1.235 |  |
| 59268 | inositol hexaphosphate kinase 2 | 1.232 | apo, growth |
| 297432 | ankyrin repeat and BTB (POZ) domain containing 1 | 1.189 |  |
| 79011 | CaM kinase-like vesicle-associated | 1.177 | sig |

id = identifier, ID = infected and dexamethasone treated animals, IS = infected and saline treated animals, GO = Gene Ontology, Apo = apoptosis, inf = inflammation, IS dev = immune system development, sig = signalling. Fold changes smaller than one indicate downregulation, and fold changes larger than one indicate upregulation
