# Supplementary material for: Adjunctive Dexamethasone Affects the Expression of Genes Related to Inflammation, Neurogenesis and Apoptosis in Infant Rat Pneumococcal Meningitis
Source: PLoS One. 2011 Mar 11;6(3):e17840. doi: 10.1371/journal.pone.0017840 (PMC3055894; doi:10.1371/journal.pone.0017840)
Supplement: Table S2 — Microarray results of significantly different genes when comparing samples of the cortex of infected and dexamethasone-treated animalswith infected and saline-treated animals (ID vs. IS). (DOC) [file pone.0017840.s002.doc]

**Supporting table S2. Microarray results of significantly different genes when comparing samples of the cortex.**

| **Entrez Gene id** | **Gene description** | **Fold change ID/IS** | **Categories of GO clusters** |
| --- | --- | --- | --- |
| 498335 | chemokine (C-X-C motif) ligand 13 | 0.120 | IS dev |
| 171164 | guanylate nucleotide binding protein 2 | 0.276 |  |
| 117029 | chemokine (C-C motif) receptor 5 | 0.332 | growth |
| 24575 | myxovirus (influenza virus) resistance 1 | 0.343 |  |
| 246208 | interferon gamma inducible protein 47 | 0.360 |  |
| 24615 | S100 calcium-binding protein A4 | 0.370 |  |
| 316137 | EGF-like module containing, mucin-like, hormone receptor-like sequence 1 | 0.384 |  |
| 304966 | Fc fragment of IgG, low affinity IIIa, receptor | 0.403 | inf |
| 171056 | chemokine (C-X3-C) receptor 1 | 0.414 | growth, inf |
| 303090 | immunity-related GTPase family, M | 0.426 |  |
| 25291 | annexin A3 | 0.429 |  |
| 25285 | insulin-like growth factor binding protein 5 | 0.429 | growth |
| 292060 | interferon regulatory factor 8 | 0.436 | IS dev |
| 497942 | chemokine (C-X-C motif) ligand 16 | 0.437 | growth |
| 114553 | neutrophil cytosolic factor 1 | 0.447 | apo, growth |
| 24387 | glial fibrillary acidic protein | 0.451 |  |
| 25423 | cathepsin C | 0.455 |  |
| 114091 | ficolin B | 0.464 | inf |
| 54320 | podoplanin | 0.471 | growth, inf |
| 287435 | Cd68 molecule | 0.479 |  |
| 292594 | leukocyte immunoglobulin-like receptor, subfamily B, member 4 | 0.479 |  |
| 366988 | bridging integrator 2 | 0.482 |  |
| 24699 | protein tyrosine phosphatase, receptor type, C | 0.482 | sig, inf, IS dev |
| 303735 | ring finger protein 213 | 0.482 |  |
| 366957 | RAS-related C3 botulinum substrate 2 | 0.484 |  |
| 24968 | proteasome (prosome, macropain) subunit, beta type 8 (large multifunctional peptidase 7) | 0.494 |  |
| 64552 | macrophage expressed gene 1 | 0.496 |  |
| 304549 | 2'-5' oligoadenylate synthetase-like 2 | 0.498 |  |
| 308995 | integrin alpha L | 0.502 | sig, inf |
| 80841 | fatty acid binding protein 7, brain | 0.502 |  |
| 298199 | adipose differentiation related protein | 0.509 |  |
| 362792 | phospholipase D family, member 4 | 0.516 |  |
| 113955 | glycoprotein (transmembrane) nmb | 0.523 | inf |
| 171452 | RAB3A interacting protein (rabin3)-like 1 | 0.523 |  |
| 362456 | Rho, GDP dissociation inhibitor (GDI) beta | 0.524 |  |
| 89783 | lysosomal-associated protein transmembrane 5 | 0.528 |  |
| 58919 | cyclin D1 | 0.530 | sig |
| 297077 | transmembrane protein 176A | 0.531 |  |
| 171411 | transmembrane protein 176B | 0.533 |  |
| 25383 | apolipoprotein B editing complex 1 | 0.534 |  |
| 315348 | NCK associated protein 1 like | 0.538 |  |
| 303378 | schlafen 8 | 0.541 |  |
| 155918 | lymphocyte cytosolic protein 2 | 0.549 | inf |
| 25124 | signal transducer and activator of transcription 1 | 0.553 | sig |
| 29591 | transforming growth factor, beta receptor 1 | 0.556 | growth, sig, IS dev |
| 25441 | Fc fragment of IgE, high affinity I, receptor for; gamma polypeptide | 0.561 | growth, inf |
| 288077 | hematopoietic cell specific Lyn substrate 1 | 0.564 | sig, IS dev |
| 81756 | RAB13, member RAS oncogene family | 0.569 |  |
| 113940 | glia maturation factor, gamma | 0.577 |  |
| 406864 | chloride intracellular channel 1 | 0.586 |  |
| 24811 | transporter 1, ATP-binding cassette, sub-family B (MDR/TAP) | 0.591 | inf |
| 302248 | nidogen 2 | 0.595 | inf |
| 155183 | src family associated phosphoprotein 2 | 0.597 | inf |
| 59073 | latexin | 0.603 |  |
| 25181 | biglycan | 0.608 | growth |
| 81515 | Yamaguchi sarcoma viral (v-yes-1) oncogene homolog | 0.620 | sig, inf, IS dev |
| 29345 | serine (or cysteine) peptidase inhibitor, clade H, member 1 | 0.621 |  |
| 25737 | proliferating cell nuclear antigen | 0.625 |  |
| 29143 | granulin | 0.635 | growth |
| 306071 | lymphocyte cytosolic protein 1 | 0.635 |  |
| 25668 | CD38 antigen | 0.637 | growth, inf |
| 295588 | Rho family GTPase 3 | 0.638 |  |
| 361991 | S100 calcium binding protein A16 | 0.642 |  |
| 58982 | neurocan | 0.645 | inf |
| 84398 | complement component 1, q subcomponent, receptor 1 | 0.646 | inf |
| 25059 | hexokinase 2 | 0.648 | sig |
| 81521 | moesin | 0.649 | growth, inf |
| 29186 | CD63 antigen | 0.652 | growth |
| 63865 | legumain | 0.653 | growth |
| 307403 | colony stimulating factor 1 receptor | 0.656 | sig |
| 310392 | solute carrier family 7 (cationic amino acid transporter, y+ system), member 11 | 0.657 |  |
| 361383 | CD97 antigen | 0.658 |  |
| 54301 | solute carrier family 14 (urea transporter), member 1 | 0.663 |  |
| 308906 | similar to 9230105E10Rik protein | 0.665 |  |
| 245962 | CD48 antigen | 0.667 | inf |
| 293650 | T-cell, immune regulator 1, ATPase, H+ transporting, lysosomal V0 protein A3 | 0.670 | sig |
| 282817 | PYD and CARD domain containing | 0.670 | apo, growth |
| 290741 | dCMP deaminase | 0.680 |  |
| 24842 | transformation related protein 53 | 0.684 | apo, growth, sig, inf, IS dev |
| 54319 | villin 2 | 0.690 | inf |
| 116685 | lamin B1 | 0.692 |  |
| 54259 | inositol polyphosphate-5-phosphatase D | 0.694 | apo, inf |
| 24404 | glutathione peroxidase 1 | 0.694 | apo, growth, sig |
| 297785 | similar to Glyceraldehyde-3-phosphate dehydrogenase (GAPDH) | 0.694 | apo |
| 25490 | neural precursor cell expressed, developmentally down-regulated gene 8 | 0.700 |  |
| 64303 | profilin 1 | 0.701 |  |
| 83628 | CD82 antigen | 0.703 |  |
| 315500 | endonuclease/exonuclease/phosphatase family domain containing 1 | 0.703 |  |
| 289014 | MAP kinase-activated protein kinase 2 | 0.705 | sig |
| 305604 | epidermal growth factor-containing fibulin-like extracellular matrix protein 1 | 0.706 |  |
| 116484 | thioredoxin 1 | 0.710 | growth |
| 54227 | actin related protein 2/3 complex, subunit 1B | 0.711 | growth |
| 140725 | calcium channel, voltage-dependent, gamma subunit 4 | 0.712 |  |
| 289668 | leucine aminopeptidase 3 | 0.717 |  |
| 360903 | fibroblast growth factor receptor-like 1 | 0.723 |  |
| 25625 | tumor necrosis factor receptor superfamily, member 1a | 0.725 | apo, sig |
| 81663 | guanine nucleotide binding protein, alpha 12 | 0.726 |  |
| 317218 | integral membrane protein 2A | 0.728 |  |
| 293118 | prolylcarboxypeptidase (angiotensinase C) | 0.730 |  |
| 54231 | carbonic anhydrase 2 | 0.734 | growth, IS dev |
| 498333 | BMP-2 inducible kinase | 0.738 | sig |
| 308444 | Axl receptor tyrosine kinase | 0.746 | growth, sig |
| 282838 | GM2 ganglioside activator protein | 0.750 |  |
| 25359 | thymopoietin | 0.751 |  |
| 690899 | platelet receptor Gi24 | 0.752 |  |
| 81513 | ligase I, DNA, ATP-dependent | 0.761 |  |
| 25406 | CD44 antigen | 0.766 | growth, inf |
| 84575 | fatty acid desaturase 1 | 0.766 |  |
| 312705 | complement component 1, r subcomponent | 0.771 |  |
| 494338 | tripartite motif-containing 25 | 0.772 |  |
| 83718 | chloride intracellular channel 4 (mitochondrial) | 0.773 | growth |
| 313130 | peptidase M20 domain containing 2 | 0.776 |  |
| 116502 | BCL2-antagonist/killer 1 | 0.778 | apo, growth, inf, IS dev |
| 292156 | SH3-domain GRB2-like B1 (endophilin) | 0.780 | apo |
| 79255 | activating transcription factor 4 | 0.785 |  |
| 296466 | Bwk1 leukemia-related gene | 0.786 |  |
| 24356 | v-ets erythroblastosis virus E26 oncogene homolog 1 (avian) | 0.787 | growth |
| 360697 | interferon gamma receptor 2 | 0.795 |  |
| 24399 | glutamate dehydrogenase 1 | 0.796 | growth |
| 300689 | REX2, RNA exonuclease 2 homolog (S. cerevisiae) | 0.811 |  |
| 360678 | Rho GDP dissociation inhibitor (GDI) alpha | 0.812 |  |
| 288778 | proliferation-associated 2G4 | 0.812 |  |
| 25097 | phospholipase D2 | 0.826 | growth, sig, inf |
| 29214 | tubulin, beta 5 | 0.832 |  |
| 64701 | ribophorin II | 0.836 |  |
| 499022 | quaking | 0.846 | growth |
| 313806 | ring finger protein 19B | 0.848 |  |
| 83783 | sulfotransferase family 1A, phenol-preferring, member 1 | 7.051 |  |
| 361734 | membrane-spanning 4-domains, subfamily A, member 4A | 5.858 |  |
| 60666 | glycerol-3-phosphate dehydrogenase 1 (soluble) | 3.512 | sig |
| 64345 | hypoxia inducible factor 3, alpha subunit | 3.420 |  |
| 24450 | 3-hydroxy-3-methylglutaryl-Coenzyme A synthase 2 | 3.225 |  |
| 361810 | FK506 binding protein 5 | 2.847 |  |
| 84403 | gap junction membrane channel protein beta 6 | 2.755 | apo |
| 65164 | HtrA serine peptidase 1 | 2.623 | growth |
| 295228 | G patch domain containing 4 | 2.487 |  |
| 408223 | ubiquitin specific peptidase 54 | 2.236 |  |
| 24484 | insulin-like growth factor binding protein 3 | 2.223 | growth |
| 303786 | apoptosis-inducing factor, mitochondrion-associated 3 | 2.206 | apo |
| 117240 | hephaestin | 2.143 | IS dev |
| 83514 | TSC22 domain family 3 | 2.069 |  |
| 25116 | hydroxysteroid 11-beta dehydrogenase 1 | 2.063 |  |
| 24701 | phosphorylase, glycogen, muscle | 2.041 |  |
| 497811 | xanthine dehydrogenase | 1.922 |  |
| 54250 | fibroblast growth factor 2 | 1.883 | apo, growth, sig |
| 353227 | zinc finger and BTB domain containing 16 | 1.856 | IS dev |
| 246246 | patatin-like phospholipase domain containing 7 | 1.851 |  |
| 312382 | ATP-binding cassette, sub-family G (WHITE), member 2 | 1.778 |  |
| 313219 | zinc finger protein 189 | 1.769 |  |
| 24539 | lipoprotein lipase | 1.765 |  |
| 24191 | aldolase C | 1.725 | apo |
| 81526 | nephroblastoma overexpressed gene | 1.723 | growth |
| 25420 | crystallin, alpha B | 1.666 | growth |
| 65037 | c-mer proto-oncogene tyrosine kinase | 1.656 | sig |
| 94174 | tubulointerstitial nephritis antigen-like 1 | 1.656 |  |
| 361749 | interleukin 33 | 1.656 |  |
| 24250 | cystathionine beta synthase | 1.655 | sig |
| 60423 | solute carrier family 28 (sodium-coupled nucleoside transporter), member 2 | 1.645 |  |
| 363711 | similar to Ciliary dynein heavy chain 9 (Axonemal beta dynein heavy chain 9) | 1.632 |  |
| 24716 | ret proto-oncogene | 1.613 | growth, sig, inf |
| 316275 | progestin and adipoQ receptor family member VIII | 1.604 |  |
| 140942 | DNA-damage-inducible transcript 4 | 1.596 | apo |
| 29610 | transforming growth factor, beta receptor III | 1.578 | growth, sig, IS dev |
| 65034 | PDZ domain containing 2 | 1.573 | inf |
| 307759 | glutamic pyruvate transaminase (alanine aminotransferase) 2 (predicted) | 1.565 |  |
| 684245 | similar to peptidylprolyl isomerase (cyclophilin)-like 6 | 1.555 |  |
| 29624 | arachidonate 5-lipoxygenase activating protein | 1.539 |  |
| 24179 | angiotensinogen (serpin peptidase inhibitor, clade A, member 8) | 1.522 | growth, sig, inf |
| 688916 | hypothetical protein LOC688916 | 1.495 |  |
| 83567 | melanin-concentrating hormone receptor 1 | 1.489 | growth |
| 24783 | solute carrier family 9 (sodium/hydrogen exchanger), member 2 | 1.482 |  |
| 310803 | exostoses (multiple)-like 2 | 1.482 |  |
| 362366 | FK506 binding protein 14 | 1.476 |  |
| 29232 | chloride channel 2 | 1.474 |  |
| 25718 | insulin-like growth factor 1 receptor | 1.456 | growth, sig |
| 299923 | N-myc downstream regulated gene 1 | 1.455 | inf |
| 25566 | tropomodulin 1 | 1.453 |  |
| 116486 | SEC14-like 2 (S. cerevisiae) | 1.450 |  |
| 361433 | copine VII | 1.447 |  |
| 293449 | von Willebrand factor A domain containing 3A | 1.444 |  |
| 81632 | 4-aminobutyrate aminotransferase | 1.444 |  |
| 619549 | phosphatidic acid phosphatase type 2 domain containing 2 | 1.440 |  |
| 313729 | ERBB receptor feedback inhibitor 1 | 1.430 |  |
| 29517 | serum/glucocorticoid regulated kinase | 1.429 | apo, growth, sig |
| 81809 | transforming growth factor, beta 2 | 1.420 | apo, growth, sig, inf, IS dev |
| 117514 | thioredoxin interacting protein | 1.419 | sig |
| 29643 | synaptic vesicle glycoprotein 2c | 1.414 |  |
| 114513 | RAS protein-specific guanine nucleotide-releasing factor 2 | 1.410 |  |
| 171522 | cytochrome P450, family 2, subfamily d, polypeptide 4 | 1.401 |  |
| 60356 | cysteine sulfinic acid decarboxylase | 1.400 |  |
| 290527 | annexin A11 | 1.398 |  |
| 50646 | protein tyrosine kinase 2 beta | 1.396 | growth, sig, inf |
| 94172 | solute carrier family 27 (fatty acid transporter), member 1 | 1.396 | growth |
| 293950 | similar to hypothetical protein | 1.395 |  |
| 170955 | zinc finger protein 483 | 1.394 |  |
| 245977 | G protein-coupled receptor 116 | 1.393 |  |
| 691922 | 5'-nucleotidase domain containing 3 | 1.390 |  |
| 171114 | N-myc downstream regulated gene 2 | 1.383 |  |
| 29660 | pregnancy upregulated non-ubiquitously expressed CaM kinase | 1.381 | sig |
| 56817 | Kv channel-interacting protein 2 | 1.378 |  |
| 64511 | farnesyltransferase, CAAX box, beta | 1.377 |  |
| 84587 | phospholipase C-like 1 | 1.377 |  |
| 114024 | acyl-CoA synthetase long-chain family member 3 | 1.372 |  |
| 364634 | CUB and Sushi multiple domains 1 | 1.371 |  |
| 64558 | frizzled homolog 4 (Drosophila) | 1.368 |  |
| 25666 | diacylglycerol kinase, gamma | 1.364 |  |
| 59326 | Rap guanine nucleotide exchange factor (GEF) 3 | 1.363 |  |
| 24380 | glutamic acid decarboxylase 2 | 1.354 |  |
| 24805 | synaptotagmin II | 1.354 |  |
| 315189 | proline rich 5 (renal) | 1.354 |  |
| 25743 | potassium inwardly-rectifying channel, subfamily J, member 6 | 1.353 |  |
| 171410 | acyl-CoA synthetase bubblegum family member 1 | 1.352 | apo |
| 79431 | basic helix-loop-helix domain containing, class B2 | 1.347 | growth |
| 81708 | aldehyde dehydrogenase family 6, subfamily A1 | 1.345 |  |
| 25450 | gamma-aminobutyric acid (GABA-A) receptor, subunit beta 1 | 1.341 |  |
| 307956 | RNA binding motif protein 34 | 1.338 |  |
| 25400 | calcium/calmodulin-dependent protein kinase II alpha | 1.331 | growth, sig |
| 298504 | major facilitator superfamily domain containing 2 | 1.330 |  |
| 81821 | diacylglycerol kinase zeta | 1.322 |  |
| 314384 | ribosomal protein S6 kinase, polypeptide 5 | 1.318 | sig |
| 498545 | TSC22 domain family, member 1 | 1.311 |  |
| 306013 | leucine-rich repeat LGI family, member 3 | 1.310 |  |
| 29358 | neuropeptide Y receptor Y1 | 1.309 | growth |
| 24791 | secreted acidic cysteine rich glycoprotein | 1.300 |  |
| 498728 | similar to Elongation of very long chain fatty acids protein 2 | 1.299 |  |
| 362235 | similar to chromosome 20 open reading frame 39 | 1.298 |  |
| 303823 | similar to mitogen-activated protein kinase kinase kinase 13; leucine zipper-bearing kinase | 1.297 | sig |
| 300836 | similar to hypothetical protein MGC38960 | 1.297 |  |
| 312440 | jumonji domain containing 1A | 1.291 |  |
| 309419 | phosphatidylinositol-4-phosphate 5-kinase, type 1, beta | 1.291 |  |
| 117560 | Kruppel-like factor 9 | 1.290 | growth |
| 293489 | GIY-YIG domain containing 2 | 1.289 |  |
| 245956 | sodium channel, voltage-gated, type III, beta | 1.288 |  |
| 25435 | eukaryotic elongation factor-2 kinase | 1.287 | sig |
| 288109 | SID1 transmembrane family, member 1 | 1.282 |  |
| 287422 | period homolog 1 (Drosophila) | 1.282 |  |
| 365865 | phosphatidylinositol-4-phosphate 5-kinase, type 1, alpha | 1.282 |  |
| 298801 | CAP-GLY domain containing linker protein family, member 4 | 1.280 |  |
| 363085 | carbonic anyhydrase 12 | 1.279 |  |
| 140866 | diacylglycerol kinase, alpha | 1.278 |  |
| 59085 | argininosuccinate lyase | 1.272 |  |
| 290566 | oxoglutarate dehydrogenase-like | 1.271 |  |
| 81530 | pyruvate dehydrogenase kinase, isoenzyme 2 | 1.270 | sig |
| 305571 | UDP-GlcNAc:betaGal beta-1,3-N-acetylglucosaminyltransferase 2 | 1.268 | growth |
| 361309 | polyadenylate-binding protein interacting protein 2 | 1.261 |  |
| 65047 | slit homolog 1 (Drosophila) | 1.260 | growth |
| 499010 | spectrin repeat containing, nuclear envelope 1 | 1.260 |  |
| 307839 | zinc finger protein 23 (KOX 16) | 1.260 |  |
| 25054 | neurotrophic tyrosine kinase, receptor, type 2 | 1.255 | growth, sig |
| 81651 | chondroitin sulfate proteoglycan 4 | 1.250 | growth, sig |
| 306204 | filamin, beta | 1.250 |  |
| 289392 | plexin A2 | 1.236 | growth |
| 60668 | amphiphysin | 1.236 |  |
| 313672 | similar to CG11206-PA | 1.233 |  |
| 311337 | leukocyte receptor tyrosine kinase | 1.228 |  |
| 361084 | LIM domain only 7 | 1.228 | inf |
| 246047 | calcium binding and coiled coil domain 1 | 1.220 |  |
| 300083 | solute carrier family 25 (mitochondrial carrier; peroxisomal membrane protein, 34kDa), member 17 | 1.220 |  |
| 309828 | TSPY-like 4 | 1.219 |  |
| 84009 | kalirin, RhoGEF kinase | 1.198 | sig |
| 680445 | muscleblind-like 2 | 1.197 |  |
| 63840 | period homolog 2 (Drosophila) | 1.186 |  |
| 363058 | cell adhesion molecule 1 | 1.184 | growth, inf |
| 308579 | carnitine palmitoyltransferase 1c | 1.169 |  |

id = identifier, ID = infected and dexamethasone treated animals, IS = infected and saline treated animals, GO = Gene Ontology, Apo = apoptosis, inf = inflammation, IS dev = immune system development, sig = signalling. Fold changes smaller than one indicate downregulation, and fold changes larger than one indicate upregulation
