# Supplementary material for: Adjunctive Dexamethasone Affects the Expression of Genes Related to Inflammation, Neurogenesis and Apoptosis in Infant Rat Pneumococcal Meningitis
Source: PLoS One. 2011 Mar 11;6(3):e17840. doi: 10.1371/journal.pone.0017840 (PMC3055894; doi:10.1371/journal.pone.0017840)
Supplement: Table S3 — Results of all genes used on TaqMan Low Density Arrays. (DOC) [file pone.0017840.s003.doc]

**Supporting table S3. Results of all genes used on TaqMan Low Density Arrays.**

| **Gene description (Gene symbol)** | **TaqMan**  **Assay id** | **Reference Sequence** | **Fold change qPCR ID/IS** | **CI (95%) qPCR ID/IS** | **Fold change MA ID/IS** |
| --- | --- | --- | --- | --- | --- |
| **Reference genes** | | | | | |
| Ribosomal protein L24 (Rpl24) | Rn00821104_g1 | NM_022515.1 | 0.923 | 0.849-0.997 | na |
| Transmembrane protein 111 (Tmem111) | Rn01425867_m1 | NM_001008355.1 | 1.033 | 0.971-1.094 | 1.010 |
| Vesicle docking protein USO1 homolog (USO1) | Rn00571385_m1 | NM_019379.1 | 1.051 | 0.986-1.115 | 0.929 |
| **Neuronal markers** | | | | | |
| Allograft inflammatory factor 1 (AIF-1) | Rn00567906_g1 | NM_017196.2 | 0.526 | 0.347-0.705 | 0.648 |
| Doublecortin (DCX) | Rn00584505_m1 | NM_053379.2 | 1.077 | 0.815-1.340 | 1.064 |
| Enolase 2, gamma (Eno2) | Rn00595017_m1 | NM_139325.1 | 1.127 | 0.884-1.371 | 1.100 |
| Glial fibrillary acidic protein (GFAP) | Rn00566603_m1 | NM_017009.2 | 0.406 | 0.269-0.544 | 0.466 |
| Nestin (Nes) | Rn00564394_m1 | NM_012987.1 | 0.760 | 0.525-0.995 | 0.701 |
| POU class 5 homeobox 1 (POU5f1) | Rn01532129_g1 | NM_001009178.1 | - | - | - |
| Tubulin, beta 3 (Tubb3) | Rn00594933_m1 | NM_139254.2 | 0.976 | 0.719-1.232 | 1.217 |
| **Neurotrophic factors and their receptors** | | | | | |
| Nerve growth factor, beta (NGF) | Rn01533872_m1 | XM_227525.3 | 1.310 | 1.125-1.495 | 1.088 |
| Brain derived neurotrophic factor (BDNF) | Rn02531967_s1 | NM_012513.3 | 1.191 | 0.945-1.436 | 1.127 |
| Neurotrophin 3 (NTF3) | Rn00579280_m1 | NM_031073.2 | 1.181 | 0.875-1.486 | 1.149 |
| Neurotrophin 5 (NTF5) | Rn00566076_s1 | NM_013184.3 | 1.119 | 0.513-1.724 | 1.034 |
| Neurotrophic tyrosine kinase, receptor, type 1 (NTRK1) | Rn00572130_m1 | NM_021589.1 | 0.731 | 0.270-1.191 | 1.014 |
| Neurotrophic tyrosine kinase, receptor, type 2 (NTRK2) | Rn01441749_m1 | NM_012731.1 | 1.380 | 1.243-1.517 | 1.171 |
| Neurotrophic tyrosine kinase, receptor, type 3 (NTRK3) | Rn00570389_m1 | NM_019248.1 | 1.191 | 1.057-1.326 | 1.042 |
| Nerve growth factor receptor (NGFR) | Rn00561634_m1 | NM_012610.1 | 0.628 | 0.447-0.810 | 1.032 |
| **Neurogenetic pathways (wnt signalling)** | | | | | |
| Catenin (cadherin associated protein), beta 1 (Ctnnb1) | Rn00584431_g1 | NM_053357.2 | 1.296 | 1.121-1.471 | 1.046 |
| Glycogen synthase kinase 3 beta (GSK3b) | Rn00583429_m1 | NM_032080.1 | 1.116 | 0.950-1.282 | 0.982 |
| Wingless-type MMTV integration site 5A (Wnt5a) | Rn01402000_m1 | NM_022631.1 | 0.928 | 0.774-1.082 | 0.873 |
| Wnt inhibitory factor 1 (Wif-1) | Rn00586968_m1 | NM_053738.1 | 1.032 | 0.849-1.215 | 1.007 |
| **Notch signalling** | | | | | |
| Notch gene homolog 1 (Drosophila) (Notch1) | Rn01758633_m1 | NM_001105721.1 | 0.968 | 0.602-1.335 | 0.964 |
| Notch gene homolog 2 (Drosophila) (Notch2) | Rn00577522_m1 | NM_024358.1 | 0.857 | 0.725-0.990 | 0.815 |
| **Neurogenetic pathways (TGF-beta signalling)** | | | | | |
| Bone morphogenetic protein 2 (BMP-2) | Rn01484736_m1 | NM_017178.1 | 0.991 | 0.547-1.435 | 0.932 |
| Inhibitor of DNA binding 1 (Id1) | Rn00562985_s1 | NM_012797.2 | 1.088 | 0.948-1.227 | 1.055 |
| MAD homolog 1 (Drosophila) (Smad1) | Rn00565555_m1 | NM_013130.2 | 1.013 | 0.751-1.275 | 0.964 |
| MAD homolog 2 (Drosophila) (Smad2) | Rn00569900_m1 | NM_019191.1 | 0.944 | 0.803-1.085 | 0.864 |
| MAD homolog 4 (Drosophila) (Smad4) | Rn00570593_m1 | NM_019275.2 | 1.068 | 0.973-1.164 | 0.996 |
| MAD homolog 5 (Drosophila) (Smad5) | Rn00572484_m1 | NM_021692.1 | 0.967 | 0.868-1.065 | 0.884 |
| Transforming growth factor, beta 1 (TGF-b1) | Rn99999016_m1 | NM_021578.2 | 0.565 | 0.445-0.686 | 0.635 |
| Transforming growth factor, beta receptor II (Tgfbr2) | Rn00579682_m1 | NM_031132.3 | 0.800 | 0.698-0.902 | 0.752 |
| **Microglial markers: OFF signals** | | | | | |
| CD47 antigen (CD47) | Rn00569914_m1 | NM_019195.2 | 1.223 | 1.115-1.332 | 1.039 |
| CD200 antigen (CD200) | Rn00580478_m1 | NM_031518.1 | 1.206 | 0.972-1.440 | 1.043 |
| Chemokine (C-X3-C motif) ligand 1 (CX3CL1) | Rn00593186_m1 | NM_134455.1 | 1.092 | 0.920-1.264 | 1.053 |
| **Microglial markers: ON signals – Chemotaxis** | | | | | |
| Chemokine (C-X-C motif) ligand 10 (CXCL10) | Rn00594648_m1 | NM_139089.1 | - | - | - |
| Purinergic receptor P2Y, G-protein coupled 12 (P2Y12) | Rn02133262_s1 | NM_022800.1 | 0.351 | 0.250-0.452 | 0.497 |
| Purinergic receptor P2X, ligand-gated ion channel 4 (P2X4) | Rn00580949_m1 | NM_031594.1 | 0.816 | 0.544-1.087 | 0.744 |
| Pyrimidinergic receptor P2Y, G-protein coupled, 6 (P2Y6) | Rn02134326_s1 | NM_057124.2 | 0.464 | 0.319-0.610 | 0.544 |
| **Matrix metalloproteinases** | | | | | |
| Matrix metallopeptidase 3 (MMP3) | Rn00591740_m1 | NM_133523.1 | 1.131 | 0.537-1.725 | 0.967 |
| Matrix metallopeptidase 13 (MMP13) | Rn01448199_m1 | XM_343345.3 | 0.966 | 0.774-1.158 | 1.015 |
| **Miscellaneous** | | | | | |
| Angiopoietin 1 (Angpt1) | Rn00585552_m1 | NM_053546.1 | 1.102 | 0.773-1.431 | 0.904 |
| Chemokine (C-X-C motif) receptor 4 (CXCR4) | Rn00573522_s1 | NM_022205.3 | 1.011 | 0.775-1.246 | 0.853 |
| Glyceraldehyde-3-phosphate dehydrogenase (GAPDH) | Rn99999916_s1 | NM_017008.3 | 1.181 | 1.042-1.320 | 1.037 |
| Phosphatase and tensin homolog (PTEN) | Rn00477208_m1 | NM_031606.1 | 1.116 | 0.992-1.239 | 1.067 |
| TSC22 domain family 3 (TSC22d3) | Rn00580222_m1 | NM_031345.1 | 2.512 | 2.095-2.929 | 1.790 |
| Vascular endothelial growth factor A (VEGFA) | Rn01511602_m1 | NM_031836.2 | 1.305 | 1.050-1.559 | 1.370 |

id = identifier, qPCR = quantitative real-time PCR, ID = infected and dexamethasone treated animals, IS = infected and saline treated animals, CI = confidence interval, MA = microarray, “-“ indicates genes that showed a coefficient of variance of the raw Cq values greater than 4 %. na: No fold change of Rpl24 was found within the MA data. A fold change below one shows downregulation, a fold change above one shows upregulation
